# Adapting to an Uncertain World: Cognitive Capacity and Causal Reasoning with Ambiguous Observations

**DOI:** 10.1371/journal.pone.0140608

**Published:** 2015-10-15

**Authors:** Yiyun Shou, Michael Smithson

**Affiliations:** Research School of Psychology, The Australian National University, Canberra, Australian Capital Territory, Australia; Center for BrainHealth, University of Texas at Dallas, UNITED STATES

## Abstract

Ambiguous causal evidence in which the covariance of the cause and effect is partially known is pervasive in real life situations. Little is known about how people reason about causal associations with ambiguous information and the underlying cognitive mechanisms. This paper presents three experiments exploring the cognitive mechanisms of causal reasoning with ambiguous observations. Results revealed that the influence of ambiguous observations manifested by missing information on causal reasoning depended on the availability of cognitive resources, suggesting that processing ambiguous information may involve deliberative cognitive processes. Experiment 1 demonstrated that subjects did not ignore the ambiguous observations in causal reasoning. They also had a general tendency to treat the ambiguous observations as negative evidence against the causal association. Experiment 2 and Experiment 3 included a causal learning task requiring a high cognitive demand in which paired stimuli were presented to subjects sequentially. Both experiments revealed that processing ambiguous or missing observations can depend on the availability of cognitive resources. Experiment 2 suggested that the contribution of working memory capacity to the comprehensiveness of evidence retention was reduced when there were ambiguous or missing observations. Experiment 3 demonstrated that an increase in cognitive demand due to a change in the task format reduced subjects’ tendency to treat ambiguous-missing observations as negative cues.

## Introduction

Inferences about causal relations require the observation and integration of causal cues, and rely on the quality of the observed evidence. In real life situations, information is often imperfect or incomplete. People are sensitive to incomplete and ambiguous information in judgment and decision making [1,2]. People may apply their knowledge to process ambiguous information in causal reasoning. People are able to infer causes that are not observed, and distinguish these hidden causes from coincidences [3,4]. Some recent studies indicate that young children can resolve ambiguous evidence when inferring causal structures (i.e., whether there is a causal link between a particular cause and an effect) by applying observed evidence such as the base rate of the effect [5,6].

In the present paper, we will address ambiguous observations that may influence reasoning about causal strength, which refers to strength of the causal link between a cause and an effect [7]. We define ambiguous information as observations where the occurrence of cause and/or effect is unclear in the sense that each observation has two or more possible states [8,9]. This definition is compatible with the judgment and decision making literature’s usage (usually referring to multiple possible values of a numerical estimate, such as a probability) and standard usage in philosophy, which can be traced back to the classical work of Black [10]. Ambiguity in observations can be manifested in different ways, such as a stimulus located at an intermediate position on a continuum that nevertheless requires categorization (e.g., whether a gray object should be classed as “dark” or “light”), or containing incomplete information that results in it occupying multiple possible states or categories (e.g., whether “hot food” refers to the food’s temperature or spiciness).

The crucial point here is that ambiguity is not a property of a stimulus, but instead a property of the inferences that judges make about the stimulus. Thus, the absence of information does not constitute ambiguity. Missing information gives rise to ambiguity only if it results in a judge inferring multiple possible states or values on the basis of that absence.

To date, there is a lack of research on causal reasoning from covariance information involving ambiguous information. Marsh and Ahn [11] conducted pioneering work testing the effects of different manifestations of ambiguity. Subjects in the experiments were presented with a sequence of pairs of stimuli. Each pair consisted of a stimulus that indicated the presence or absence of an effect, and a cause stimulus. The presence of the cause was represented by a long line, while its absence was represented by a short line. In Experiment 1, a proportion of the well-defined cause stimuli were replaced by ambiguous observations which were manifested in one of two ways. The first used lines with intermediate lengths, while the second used stimuli labelled as “unknown”. Both types of observations entailed ambiguity, as the cause could be viewed as either present or absent.

Subjects were asked to estimate the frequencies with which cause and effect covaried, and the strength of the causal associations in each condition. Marsh and Ahn [11] found that in the conditions with intermediate causal stimuli, subjects’ estimates of the frequencies of covariation events were significantly higher than the frequencies of those well-defined covariation stimuli. However, for the “unknown” causal stimuli, subjects’ estimates of the frequencies of covariation did not significantly differ from the well-defined stimuli. Marsh and Ahn suggested that subjects might not explicitly incorporate the “unknown” stimuli in their causal judgments, as a result of feeling unjustified to make the assimilation.

An alternative explanation for their results is that subjects may have interpreted their task as estimating the frequency of covariation only when the statuses of both cause and effect were known, thereby including stimuli with the intermediate state, but excluding stimuli labeled as “unknown”. Thus, it is possible that subjects incorporated the unknown stimuli when inferring causal strength. However, because there were no conditions wherein the unknown stimuli were absent or replaced by unambiguous stimuli, it is unclear whether people treated the unknown stimuli as positive or negative cues for the associations between cause and effect, or whether they simply ignored those stimuli entirely.

In addition, there is still a general lack of understanding of the cognitive mechanism of causal reasoning with “ambiguous-unknown” (AU) observations. We denote the unknown stimuli (those in which it is not known whether the effect is present or absent) by the label “ambiguous-unknown” to remind readers that in our framework, absent information results in judges inferring ambiguous (i.e., multiple possible) states. It has been shown that cognitive resources such as memory and computational capacities are essential to underlying reasoning processes that involve holding and evaluating evidence [12]. Pushkarskaya, Liu, Smithson, and Joseph [8] revealed that evaluating ambiguous information is associated with the activation of brain areas that had previously been found to be used for sample space partitioning, probability evaluations, and numerical information integration. They suggested that processing ambiguous information may involve deliberative processing. This implies that causal reasoning with ambiguous information may require additional cognitive resources to those required for reasoning with unambiguous information.

The studies reported here focused on ambiguous information in the form of unknown observations, and aimed to explore how treatment of the AU observations might be influenced by cognitive capacities. Firstly, we tested whether subjects ignore the AU (the unknown) observations in causal reasoning, as suggested by Marsh and Ahn. In Experiment 1, we compared the causal ratings of subjects in a condition that included AU stimuli with a condition where the AU observations were omitted. If subjects ignored AU observations in their causal strength estimations, then the causal ratings in the AU condition should not differ from the omission condition. In addition, we aimed to explore how subjects might treat the AU observations. We compared subjects’ causal ratings of the AU condition with a condition where the AU observations were replaced by unambiguous observations, with the two alternative states distributed equally.

We examined two plausible hypotheses regarding the treatment of AU observations. We first hypothesized that subjects might regard AU observations as equally likely to possess either of the two possible statuses. The time-honoured Principle of Indifference is one argument in favor of this hypothesis, and so is the empirical evidence from studies of partitioning effects [13], suggesting that people anchor their probability judgments on the number of alternative outcomes (e.g., if there are two alternatives, people will anchor on probabilities of 1/2). The second hypothesis was that subjects would base their probability judgments regarding the AU observations on the base-rate of the unambiguous observations available to them at the time. In our experimental setup, if subjects treated the AU observations as equally likely to have either of the two possible statuses, their ratings in the AU condition should not be significantly different from the unambiguous condition.

Both Experiment 2 and Experiment 3 examined how the processing of AU observations can be influenced by the availability of cognitive resources for retaining, evaluating and integrating AU observations during causal reasoning. The availability of cognitive resources can be determined both internally by one’s cognitive capacities and externally by demands from the environment.

The quality of data acquisition and the ability to apply rule-based reasoning can be influenced by subjects’ cognitive capacities for retaining and processing observations [14–17]. Working memory capacity (WMC) is a well-known indicator of cognitive capacity. WMC has been found to be associated with the depth of evidence encoding [18] and the ability to retain rapidly-presented evidence [19]. According to the most recent theoretical frameworks for working memory (WM), one key element of WMC is the ability to simultaneously store and process information [20,21,22]. Oberauer et al [21] defined simultaneous storage and processing as the ability to retain new and briefly-presented information over a set period of time, and then either transforming that information or deriving new information from it.

If processing AU observations involves deliberative processing, the capacity of WM for simultaneous storage and processing may determine how well subjects maintain, process, and integrate AU observations in causal judgments. Experiment 2 increased subjects’ memory demand and explored the association between ambiguity and cognitive capacities that were measured by two widely-used WM measures: operation span task and single *n-*back task. We hypothesized that under high cognitive demand conditions, subjects with lower WMC would be less likely to integrate AU observations and that in comparison to subjects with higher WMC, their causal ratings would be similar to ratings in which they ignored the AU information.

Finally, external constraints can increase demand on storage and online computational resources, which in turn can affect how comprehensively subjects retain and evaluate causal observations. In Experiment 3, we compared subjects’ causal ratings under the high cognitive demand condition versus the low cognitive demand condition. We hypothesized that under a high cognitive demand condition, subjects’ causal ratings would be similar to ratings wherein they ignored AU information.

## Experiment 1

The causal reasoning task in Experiment 1 asked subjects to evaluate the causal relation between a candidate cause and effect from covariation information. The covariation information contained the frequency of the conjunction and disjunction of a cause and an effect. We employed the paradigm introduced by Buehner et al. [12] to control for the effects of cognitive demand.

### Method

#### Ethics statement

The research (that encompasses all three Experiments) has received ethics approval from the Australian National University Human Research Ethics Committee. Informed consent was obtained in writing from all participants prior to commencing their involvement in the experiment.

#### Design

Experiment 1 involved a single causal reasoning task that contained nine experimental blocks. These were comprised of three evidence types (Ambiguous-Unknown [AU] vs. Unambiguous vs. Omission) and three contingency levels (Positive vs. Zero vs. Negative). Table 1 shows how stimuli were allocated across the different trials. We presented 12.5% of the paired stimuli with AU effect events in the AU condition. The AU stimuli were removed from the evidence set in the omission condition. Thus, the probability of the presence of the effect (E) given the presence of the cause (C), *P*(E+|C+), based on the frequencies of the unambiguous observations in the AU condition, differed from the unambiguous condition. In the unambiguous condition, half of the AU stimuli were replaced by the presence of the effect; the other half were replaced by the absence of the effect. Thus, if subjects simply treated the AU information symmetrically, i.e., regarded the effect as equally likely to be present or absent, the causal ratings under the AU condition should not differ from those under the unambiguous condition.

**Table 1:** Experimental Stimuli: Frequencies of Covariance Events in Different Conditions.

Chemical	Present		Absent		Based on Unambiguous Observations			
Virus	Inactive	Active	Inactive	Active				
**UA** ^a^					**P(E+|C+)**	**P(E+|C-)**	**ΔP**	**Causal Power**
Positive	4	12	12	4	0.75	0.25	0.5	0.67
Zero	4	12	4	12	0.75	0.75	0	0
Negative	12	4	4	12	0.25	0.75	-0.5	-0.67
**PC** ^b^								
Positive	2 + 2A	10 + 2A	12	4	0.833	0.25	0.583	0.777
Zero	2 + 2A	10 + 2A	12	4	0.833	0.75	0.083	0.332
Negative	10 + 2A	2 + 2A	4	12	0.167	0.75	-0.583	-0.777
**AC** ^c^								
Positive	4	12	10 + 2A	2 + 2A	0.75	0.167	0.583	0.700
Zero	4	12	2 + 2A	10 + 2A	0.75	0.833	-0.083	0.100
Negative	12	4	2 + 2A	10 + 2A	0.25	0.833	-0.583	-0.700

^a^ *UA* is the unambiguous condition,

^b^ *PC* is the condition where the cause was present in unknown observations,

^c^ *AC* is the condition where the cause was absent in unknown observations.

We also included the prediction of two well-known models of causal reasoning–ΔP [23] and causal power model [24]–on the causal strength if subjects estimated the causal strength with unambiguous information. Note that both ΔP and causal power model predict an increase in causal strength magnitudes if subjects omit unknown information when comparing results in the AU condition to those in the unambiguous condition.

The three contingency levels represent three different types of causal direction. A positive contingency condition imposes a generative causal link between the cause and the effect, where the probability of the effect increases in the presence of the cause. A negative contingency condition imposes a preventive causal link between the cause and the effect, where the probability of the effect decreases in the presence of the cause. Both the positive and negative contingency conditions were ambiguous regarding causal strength but not regarding causal direction. That is, it was clear whether the cause had a positive or negative effect. A zero contingency condition imposes no link between cause and effect so that the probability of the effect is the same in the presence and absence of the cause. If subjects believed that there was a causal link between the cause and effect, then both the direction and strength of causal association in this condition were unknown. Examining the effects of ambiguity in these three different contingency conditions could provide insight into how subjects treat AU information, as influenced by the nature of the link between cause and effect.

If subjects apply a single strategy across all three conditions, we should observe identical effects of ambiguity regardless of the contingency. On the other hand, if subjects are differentially sensitive to ambiguity depending on whether they believe a cause prevents or produces an effect, we should observe the effect of ambiguity being moderated by the contingency condition.

#### Participants

A total of 70 subjects were recruited via the online crowdsourcing platform Crowdflower. Fourteen subjects failed the validation test question and were excluded from the data analyses. The remaining 56 subjects (33 females; 54.3% had completed tertiary and above education) had an average age of 38.64 years (*SD* = 11.16). Subjects were paid 30 American cents for their participation.

#### Materials and procedure

Subjects were asked to pretend to be employees of a neurovirology research institution. Their task was to evaluate the effects of a range of chemicals on certain types of viruses which would cause neurological diseases. The subjects observed stimuli that indicated the status of the virus and the presence or absence of the chemical, and judged whether the chemical was an activator or inhibitor of the virus. In each experimental block 32 paired stimuli were displayed on a single screen. Subjects were shown two sets of virus samples, of which one set had not been exposed to the testing chemical (i.e., the effect status in absence of the cause), and the other had been exposed to the testing chemical (i.e., the effect status in presence of the cause). The status of a sample virus was either activated or inactivated.

The composition of the stimuli is shown in Table 1. In the AU condition, the AU outcome viruses were represented by a grayed image with a question mark in the center. The instruction preceding the AU condition was that, “For some reason, the status of some viruses was not clear; they are represented by the grayed picture with a question mark on it”. In the omission condition, these AU observations were removed from the evidence. All experimental blocks were presented to subjects in random order.

After observing the sample viruses in each experimental block, participants were asked, “How likely do you think it is that the chemical activates or inactivates this type of virus?” They rated the likelihood on a scale from -100 to 100. They were told that a negative rating meant that the chemical inactivated this type of virus; a positive rating meant that the chemical activated this type of virus; a zero rating was appropriate when they thought that there was no causal relation between the chemical and the activation of that type of virus (see S1 Appendix for more details). We acknowledge the possible influence that the scale may elicit either structure or strength judgments. However, in Shou and Smithson [25], we showed that subjects had similar responses to structure judgments and strength judgments. In the current experiment, we assumed that subjects would interpret the scale (either structure or strength) consistently. The within-subject comparison between their responses in the AU condition and the unambiguous condition still indicates how they may treat AU information.

At the end of the study, subjects were asked to indicate the strategy that best described their way to process the AU observations. They were asked to select one of the following choices: that they (a) regarded the AU virus samples as active viruses; (b) regarded the AU virus samples as inactive viruses; (c) regarded the AU virus samples as half active viruses and half inactive viruses; (d) estimated the AU observations depending on the probability of unambiguous active viruses in the presence of the chemical; (e) estimated the AU observations depending on a causal link between the unambiguous viruses and the chemical; or (f) ignored the AU viruses.

#### Data analysis

We applied beta regression analysis with Bayesian parameter estimation to examine the effects of different factors on both the mean and variability of subjects’ causal ratings [26]. The beta regression has two submodels. A location submodel models the mean and indicates how the location of the dependent variable distribution changes as a function of the predictors, while a precision submodel deals with the predictors of the precision and indicates how the variability of the dependent variable changes as a function of the predictors. Because we did not use conventional significant testing in our data analysis, we used DIC values to determine the significance of the contribution of a factor. Analyses were performed in JAGS (version 3.4.0). We took 4000 samples for obtaining model fitting values and parameter estimation values after an initial 4000 samples. For each analysis, we selected the final model based on ΔDIC (ΔDIC = DIC_model2_ –DIC _model1_ is the difference between model 2 with a factor and model 1 without that factor, indicating the DIC contribution of the factor to model 2), the contribution of a factor to the model DIC [27]. ΔDIC above a value of 3 indicates a reliable contribution of a factor to the model [27]. Any interaction terms which had ΔDIC value of less than 3 were not included in the final model. We reported the estimated coefficient and 95% credibility interval (95%CI) for each individual factor in either the location submodel or the precision submodel of the beta regression model (see S2 Appendix for details).

For readers who are not familiar with Bayesian analysis, we have also included conventional null hypothesis significance testing (NHST) results. All models were replicated by using maximum likelihood estimates (MLE). The analyses were carried out in SAS 9.4. The estimated parameter values using the MLE method were very similar to the values obtained by Bayesian analyses, thus we have only attached the NHST *p* values to each analysis.

### Hypotheses

We first examined whether subjects reasoned with the unambiguous information. We hypothesized that subjects’ ratings in the AU condition would not be significantly different from those in the omission condition if the subjects ignored the AU observations. If subjects did not ignore the AU observations, we then examined how subjects treated the AU observations by comparing the ratings in the AU condition to those in the unambiguous condition.

Our second hypothesis was that subjects’ ratings in the AU condition would not be significantly different from those in the unambiguous condition if the subjects treated the AU observations as equally likely to have produced or not produced the effect, regardless of the contingency. If subjects did not treat the AU observations symmetrically, we hypothesized that the deviance between the ratings in the AU and unambiguous conditions should be in the same direction across all three contingency conditions, and that the subjects’ causal ratings in the AU condition should be more positive than in the unambiguous condition.

### Results

**Fig 1:**
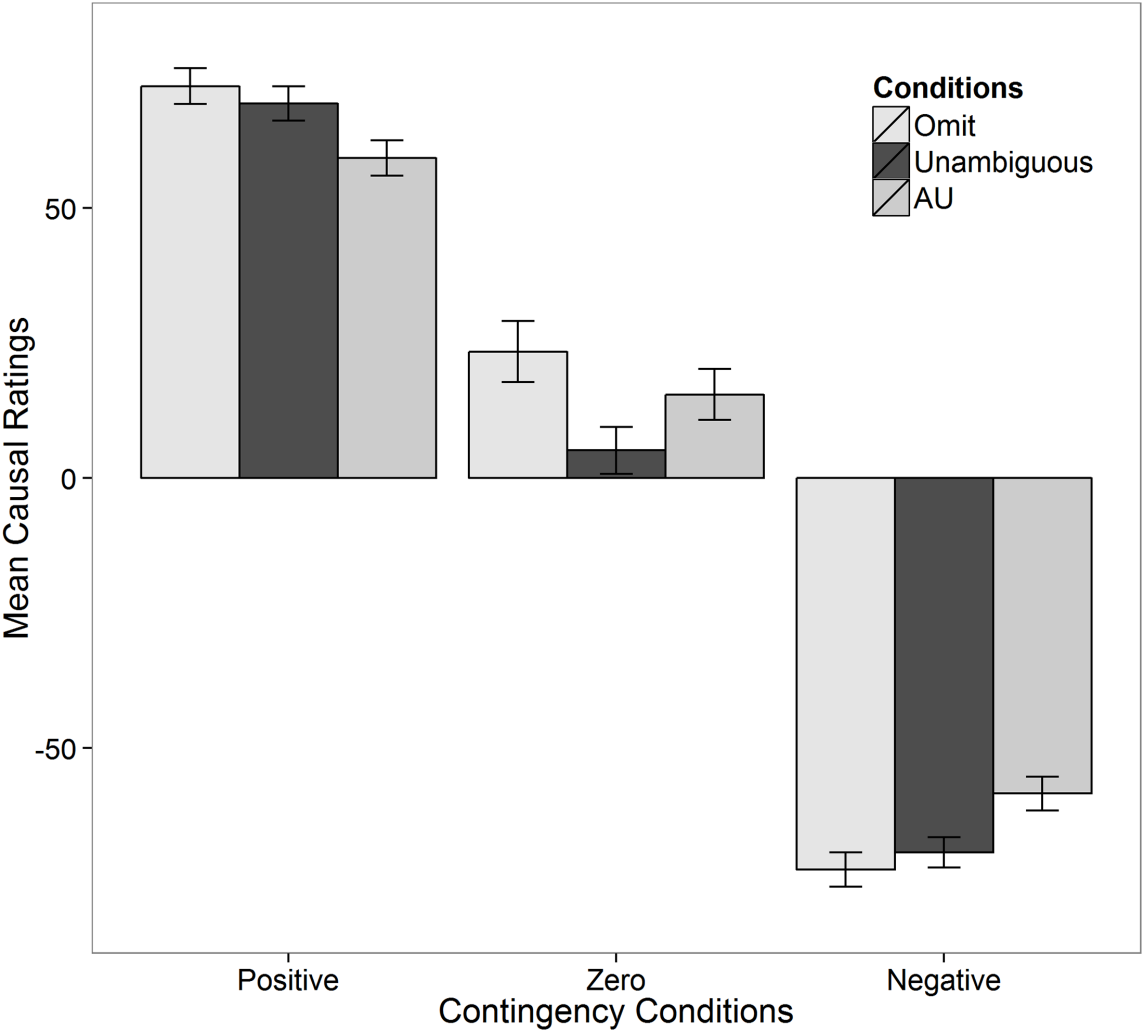
Mean of causal ratings in conditions in Experiment 1. Error bars are standard errors. AU is the ambiguous-unknown condition.

Fig 1 displays the subjects’ mean causal ratings in the different conditions. The ratings in the AU condition seem to be lower than in the omission condition for the positive and zero conditions, and less negative than those in the omission condition for the negative contingency condition. The ratings in the AU condition appear lower than those in the unambiguous condition for the positive condition, while being more positive than those in the omission condition for the zero, and less negative in the negative contingency conditions. These claims and the hypotheses are tested below.

Beta regression was conducted to examine the significance of the differences between the various conditions. Table 2 displays the results of the final model, selected on the basis of DIC values. There was a significant interaction between the ambiguity/omission conditions and contingency conditions on the mean of subjects’ ratings (ΔDIC = 15).

**Table 2:** Beta Regression on Causal Ratings Predicted by Evidence Types and Contingency Conditions.

Variables	Parameter	Coefficient	*SE*	2.5%CI	97.5%CI	*p*
**Location Submodel**						
Intercept	*b*_0_	1.40	0.12	1.16	1.64	< .001
Unambiguity	*b*_1_	0.40	0.16	0.10	0.74	.025
Omitting	*b*_2_	0.43	0.18	0.03	0.77	.020
C2	*b*_3_	-0.94	0.17	-1.04	-0.60	< .001
C3	*b*_4_	-2.77	0.17	-3.10	-2.40	< .001
Unambiguity x C2	*b*_5_	-0.81	0.22	-1.20	-0.31	.001
Unambiguity x C3	*b*_6_	-0.76	0.23	-1.29	-0.40	.002
Omitting x C2	*b*_7_	-0.30	0.25	-0.78	0.19	.230
Omitting x C3	*b*_8_	-0.90	0.23	-1.37	-0.33	< .001
**Precision Submodel**						
Intercept	*d*_0_	1.69	0.14	1.35	1.90	< .001
Unambiguity	*d*_1_	0.14	0.15	-0.15	0.58	.347
Omitting	*d*_2_	-0.06	0.15	-0.37	0.21	.702
C2	*d*_3_	-0.49	0.15	-0.56	0.00	.005
C3	*d*_4_	0.17	0.16	-0.13	0.47	.266

The dummy variable *C2* was coded as 1 for the zero contingency condition, and 0 for the other two contingency conditions; *C3* was coded as 1 for the negative contingency condition, and 0 for the other two contingency conditions. The dummy variable *Unambiguity* was coded as 1 for the unambiguous condition, and 0 for the other two conditions; *Omitting* was coded as 1 for the omitting condition, and 0 for the other two conditions. Parameter *b* are the parameters of the variable in the location submodel, while parameter *d* are the parameters of the variable in the precision submodel (see S2 Appendix for a more detailed description). A positive *b* value indicates that the mean of the condition coded as 1 is higher than the condition coded as 0. A positive *d* value indicates that the precision of the condition coded as 1 is greater than the condition coded as 0. 2.5%CI and 97.5%CI are the lower and upper bounds of the 95% Bayesian credibility interval.

The first hypothesis, that there would be no difference between the AU condition and the omission condition, was rejected. The causal ratings in the omission condition were significantly more positive than the ratings in the AU condition for the positive contingency condition (*b*_2_ = 0.43), and were slightly more positive than in the AU condition for the zero contingency condition (*b* = *b*_2_ *+ b*_7_ = 0.43–0.30 = 0.13). The causal ratings in the omission condition were significantly more negative in the omission condition than they were in the AU condition (*b* = *b*_2_ *+ b*_8_ = 0.43–0.85 = -0.42).

The second hypothesis of there being no difference between the AU condition and the unambiguous condition was also rejected. The causal ratings in the unambiguous condition were significantly more positive than the ratings in the AU condition for the positive contingency condition (*b*_1_ = 0.40), and were significantly more negative than in the AU condition for the zero contingency condition (*b* = *b*_1_ *+ b*_5_ = 0.40–0.81 = -0.41) and the negative contingency condition (*b* = *b*_1_ *+ b*_6_ = 0.40–0.76 = -0.36).

#### Post-hoc strategy selection

**Fig 2:**
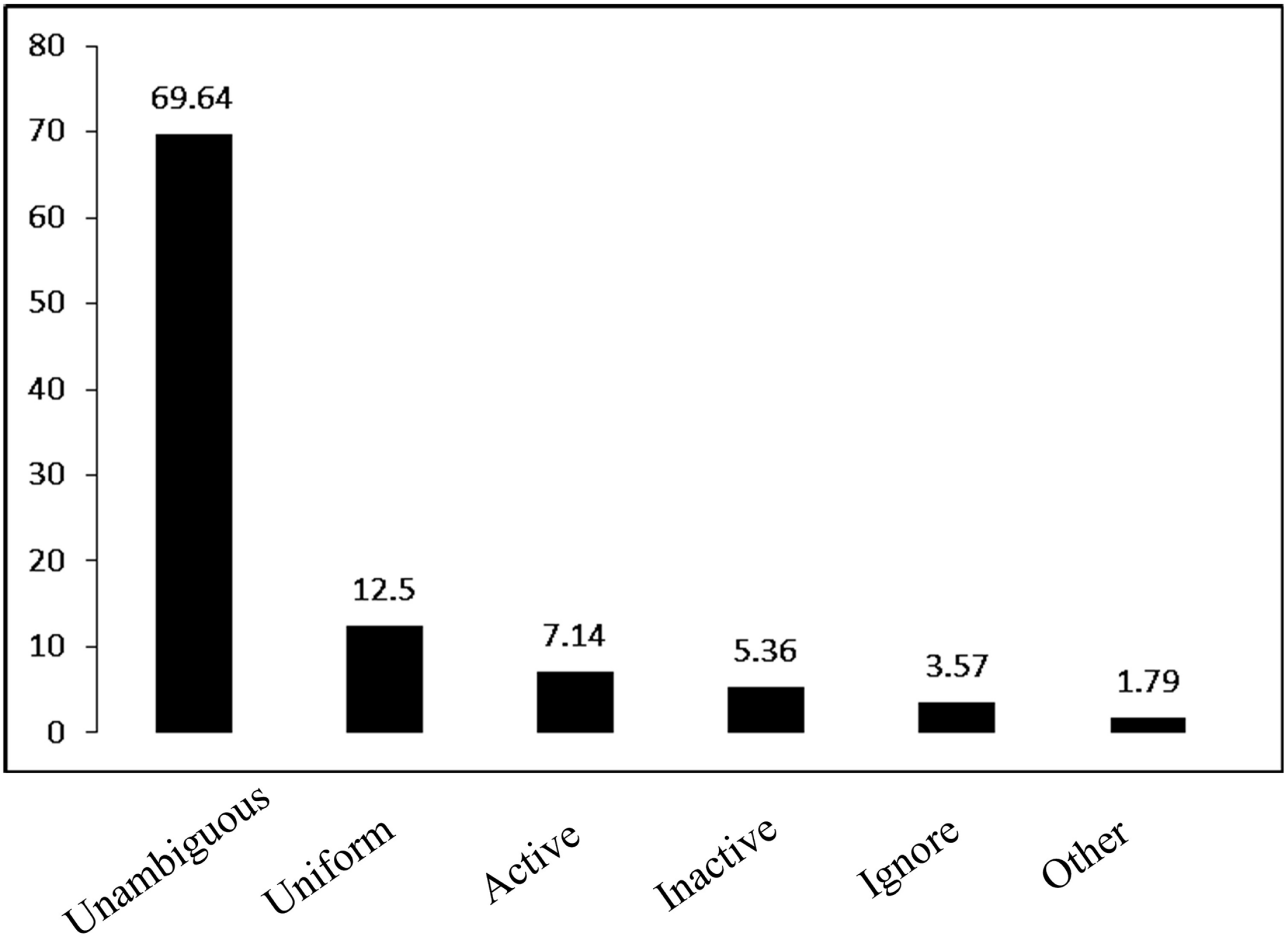
Percentages of strategy selection among subjects in Experiment 1. *Unambiguous*: estimated the AU observations depending on the probability of unambiguous active viruses, or the causal link between the unambiguous viruses and the chemical. *Uniform*: regarded the AU virus samples as equally likely to be active or inactive viruses. *Active*: regarded the AU virus samples to be active viruses. *Inactive*: regarded the AU virus samples to be inactive viruses. *Ignore*: ignored the AU samples. *Others*: other strategies.

Turning to the strategies subjects might use to estimate the AU information (see Fig 2), a majority of subjects indicated that they applied the information in the unambiguous condition to estimate the status of the AU observations. Nearly 70% of subjects indicated that that they applied the observed unambiguous information, including using current causal association or the conditional probability of the virus status. In contrast, only two subjects indicated that they ignored the AU information.

### Discussion

The first hypothesis, whereby the effects of the omitted information were significantly different from the effects of ambiguous-unknown (AU) observations on causal reasoning, was rejected, as results indicated that subjects did not merely omit the AU observations in causal reasoning. In addition, the post-hoc strategy classification suggests that 95% of subjects indicated that they applied some strategies to estimate the AU observations. Rather than applying one single imputation method (i.e., assuming all AU observations equate active/inactive viruses), subjects reported applying strategies reactively depending on the information available in the reasoning context.

The second hypothesis, that subjects treated the AU observations symmetrically (i.e., treating the AU observation as equally probable for the effect to be present or absent), was also rejected. The magnitudes of the causal ratings were significantly smaller in the AU condition than they were in the unambiguous condition in both the positive and negative contingency conditions, indicating that subjects perceived the causal links as weaker in the AU condition than in the unambiguous condition. Subjects did not appear to assume that the AU observations were equally likely to be present or absent. This finding corroborates the idea that estimating the probabilistic information under ambiguity is not symmetrical [28].

The final hypothesis, that subjects might tend to treat the AU observations as evidence for a positive contingency between the cause and effect, was also rejected. We noticed that when the direction of the causal association was not AU (i.e., in both positive and negative contingency conditions), the perceived magnitude of causal strength reduced in comparison to that in the unambiguous condition. As suggested by Garcia-Retamero and Rieskamp [29], people may be more likely to treat incomplete information as negative cues when the incomplete information is not distributed uniformly in the data. Similarly, subjects appeared to treat AU observations as negative cues against the assumption that the chemical activated/inactivated the virus, which resulted in a lower estimate of the causal strength. The significant differences in ratings between the AU and unambiguous conditions across different contingency conditions suggest that subjects treated the AU observations adaptively depending on the information available in the reasoning context.

## Experiment 2

Experiment 1 demonstrated that subjects did not merely ignore AU observations, and that they did not treat AU observations symmetrically. Experiment 2 investigated how subjects treated the AU information in a trial-by-trial task paradigm, in which subjects were presented with the observations sequentially. The sequential presentation makes greater cognitive demands on subjects than the summary format used in Experiment 1.

We observed in Experiment 1 that subjects’ causal ratings in the unambiguous condition generally had smaller magnitudes than in the omission condition, and generally greater magnitudes than in the AU condition. If treating AU observations was not influenced by cognitive resources, then the difference between the AU and unambiguous conditions should be similar to those observed in Experiment 1. The causal ratings in the AU condition would be less positive than those in the unambiguous condition for the positive contingency condition, and more positive than in the unambiguous conditions for the zero and negative contingency conditions. By contrast, if subjects were making principal-based and more cognitive demand inferences about the AU observations in Experiment 1, we would expect the divergence between the unambiguous and AU conditions to be reduced when the subjects lack the spare capacity to do this additional inference.

Next, if processing AU observations requires more processing resources, WMC in simultaneous storage and processing may determine how well subjects maintain, process, and integrate the AU observations in causal judgments. Subjects who have a lower WMC may be more likely to discard AU observations and more likely to have causal ratings with greater magnitudes in the AU condition than in the unambiguous observations.

We assessed the subjects’ WMC simultaneous storage and processing capacities by adopting two WM tests: the *n-*back task and the operation span task. Both tasks have been shown to have sound validity and reliability [20,30,31]. Both tasks also appear to capture simultaneous storage and processing, and their overlap in the cognitive processes is also reflected in brain imaging studies [32,33]. We hypothesized that subjects who scored lower in the two WM tasks would be more likely to ignore the AU observations and produce causal ratings with greater magnitudes, whereas subjects who scored higher in the two WM tasks would be more likely to integrate the AU observations as negative cues which, in turn, would lead to lower causal ratings.

### Method

#### Participants and design

A total of 72 subjects, who were first-year psychology students at Australian National University, participated in the experiment for course credit. The experiment involved a causal reasoning task and two WM tasks. We excluded one participant who pressed the wrong key in the WM test, and one participant who did not successfully complete the WM tests. The remaining 70 subjects (44 females) had an average age of 21.33 years (*SD* = 4.21). The causal reasoning task comprised of two ambiguity conditions (Ambiguous vs. Unambiguous) and three levels of contingency, a total of 12 within-subject conditions. All tasks were computer-based, and were programmed using Inquisit 4.0.

#### Materials

**Causal reasoning task:** The cover story of the task was the same as in Experiment 1. Subjects were asked to evaluate the effects of a range of chemicals on certain types of viruses. Each experimental block contained 32 pairs of stimuli which described the effects of one type of chemical on one type of virus. Each stimulus consisted of a picture of a virus on the right side of the screen with information indicating whether the virus had or had not been exposed to the testing chemical. The color of the virus was either red (activated) or blue (inactivated). If the virus had been exposed to the chemical, a chemical structure picture was displayed on the left side of the screen. If it had not been exposed to the chemical, a capitalized text stating “NO TESTING CHEMICAL” was displayed. The paired stimuli were presented sequentially. Each pair was shown onscreen for two seconds, followed by a blank screen for a one-second interval.

After observing the first 16 paired results, participants were asked to make their first judgment of the causal relation between the virus and the chemical. They were asked, “How likely do you think it is that the chemical activates or inactivates this type of virus?” The then proceeded to observe the remaining 16 paired stimuli. After observing all 32 sample viruses, the subjects rated their beliefs of the causal relations. The three blocks in each AU/unambiguous condition section were randomly presented to subjects. The order of the AU and unambiguous conditions was counter-balanced.

**Single *n*-back task:** The *n*-back test has been associated with a wide range of reasoning abilities [31]. Subjects are required to hold a representation of an item in mind while tracking stimuli which need to be compared with the representation stored in memory. Subjects need to actively update the representation after each match takes place. Attention, selection, inhibition, and memory updating are all involved in the task [30].

We applied the single *n*-back task as developed by Jaeggi, Studer-Luethi, et al. [31]. In each experimental trial, subjects were presented with a sequence of 20 letters. Subjects were required to press “A” on the keyboard when the current letter matched the one from *n*-steps earlier in the sequence. We tested 1-back, 2-back, and 3-back. Subjects were given three practice trials (one for each *n*-back) with feedback provided on their overall performance. After completing the practice trials, subjects then completed nine experimental trials: three for each *n*-back condition. The scoring of the *n*-back task depended on the total number of correct responses and the number of false alarm responses (subjects pressing the button when they should not have done so). The final score was calculated by subtracting the total number of false alarms from the total number of hits, averaged over the nine blocks.

**Operation span task:** The operation span (Ospan) task requires subjects to remember a sequence of letters while solving a range of mathematical problems. Focused attention, memory span, and active arithmetic information processing are assessed in the task [20]. The Ospan task has been demonstrated to have reasonable test-retest reliability, as well as good convergence validity with other WM measures and higher order cognition tasks [20].

We used the Ospan task as developed by Unsworth, Heitz, Schrock, and Engle [34]. At the beginning of the task, subjects were instructed to go through three practice blocks: one where they only recalled the task, one in which they only solved a mathematical question, and a final block in which they performed both tasks simultaneously. After completion of the practice blocks, the experimental trials ran combining both tasks. The length of the letter sequence ranged from 3 to 7 letters long. There were three trials for each of these sequence lengths, making 15 trials altogether using 75 letters in total. The final score used in later analysis was the total number of correctly-recalled elements from trials in which all letters were recalled in correct serial order with a simultaneous accuracy of more than 85% in solving the mathematical problems.

#### Procedure

The experimental sessions were conducted in small groups. The group sizes ranged from 5 to 12 subjects. Each subject worked individually on a computer individually. At the beginning of the experiment, the experimenter instructed the group of subjects. For the causal reasoning tasks, each subject was provided with a worksheet on which they could write down their answers and notes. Subjects were allowed to write on the worksheets only after they had observed all the stimuli in one block. Subjects first went through a self-paced practice block in which they pressed the space bar to proceed with paired stimuli. Subjects then completed the six experimental blocks, in which each pair of stimuli remained onscreen for two seconds before being automatically followed by the next pair of stimuli. After completing the causal reasoning task, participants were required to take a 5–10 minute break. They then performed the two WM tasks. There was also a 5–10 minute break between each of the two WM tasks.

### Results

**Fig 3:**
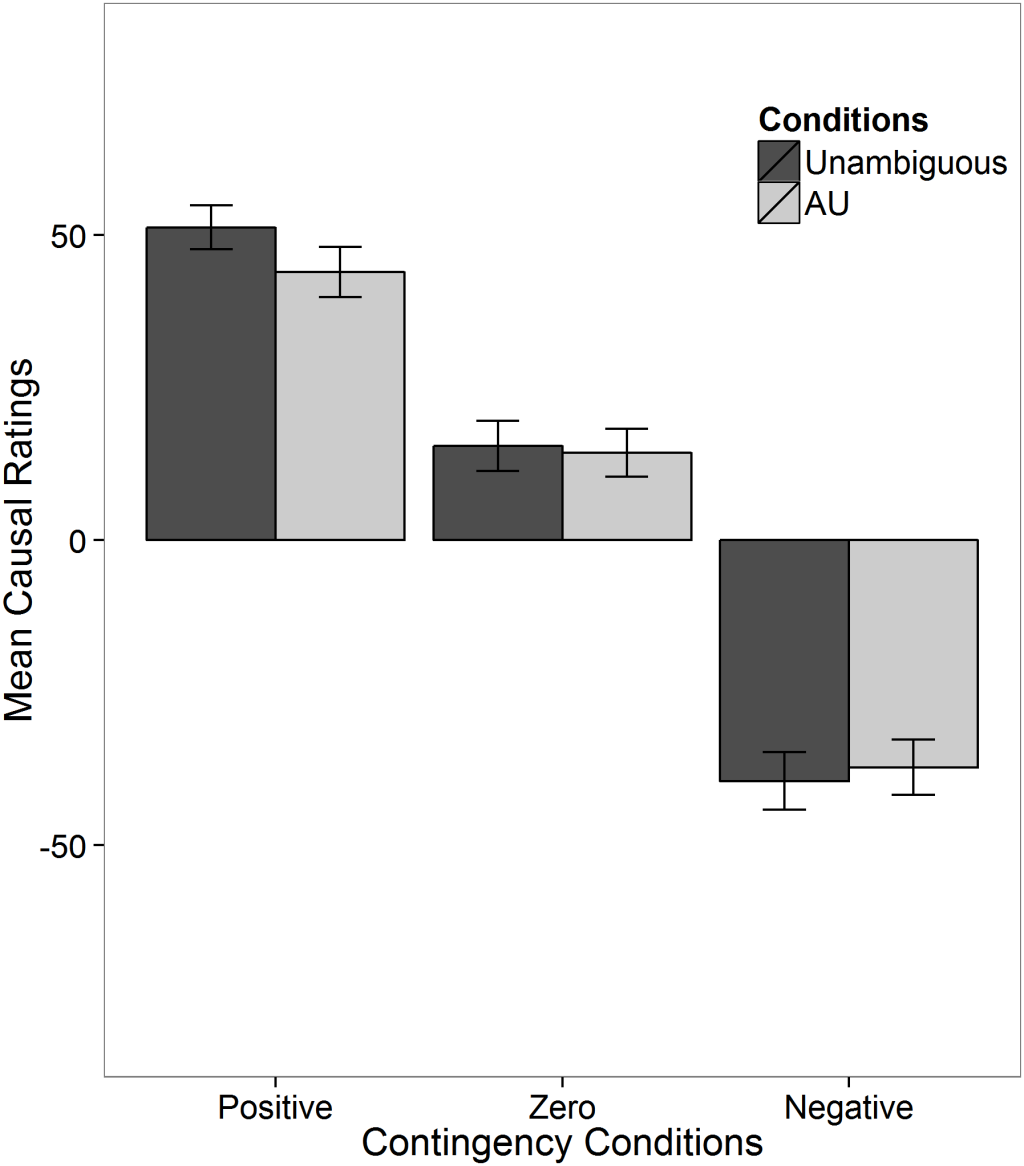
Mean causal ratings across conditions in Experiment 2. Error bars are standard errors.

The means, standardized deviations and correlations of the two WM measures are shown in Table 3. Fig 3 displays subjects’ mean causal ratings across different conditions. The pattern of the differences in the causal ratings between the AU and unambiguous condition was similar to the results found in Experiment 1, but with much smaller magnitudes.

**Table 3:** Mean, Standard Deviations and Correlations of Working Memory Measures.

	*M*	*SD*	Range	A-Ospan^a^	T-Ospan^b^
**Experiment 2**					
*n-*back	3.91	0.66	1.89–5.00	.33**	.24*
Absolute Ospan	45.59	15.55	11–75		.92***
Total Ospan	60.59	10.19	30–75		
**Experiment 3**					
*n-*back	3.48	0.70	1.75–4.88	.34**	.25
Absolute Ospan	27.46	10.43	4–50		.92***
Total Ospan	39.13	7.53	11–50		

^*a*^ *A-Ospan*: the absolute scoring of the Ospan task, the number of recalled items of the corrected recalled sequences.

^*b*^ *T-Ospan*: the total scoring of the Ospan task, the total number of items recalled across all trials.

*p < .05,

**p < .01,

***p < .001

The causal ratings received after the 32 observations for both the unambiguous and AU conditions were analyzed using beta regressions. Results of the final model are shown in Table 4. Ambiguity did not have a significant main effect on the mean or the precision of causal ratings ($Delta$ΔDICs $<$< 3). Ambiguity also did not have a significant interaction in the contingency condition ($Delta$ΔDICs $<$< 3).

**Table 4:** Effects of Ambiguity and WM measures on Causal Ratings in Experiment 2.

Variables	Parameter	Estimated	*SE*	2.5%CI	97.5%CI	*p*
**Location Submodel**						
Intercept	*b*_0_	0.16	0.04	0.09	0.24	< .001
Ambiguity	*b*_1_	-0.02	0.04	-0.10	0.08	.546
C2	*b*_2_	0.12	0.05	0.02	0.22	.015
C3	*b*_3_	-0.96	0.06	-1.09	-0.85	< .001
*n*-back	*b*_4_	0.00	0.04	-0.08	0.08	.982
Ospan	*b*_5_	0.01	0.04	-0.07	0.08	.802
**Precision Submodel**						
Intercept	*d*_0_	1.84	0.06	1.70	1.96	< .001
Ambiguity	*d*_1_	-0.00	0.06	-0.12	0.12	.988
C2	*d*_2_	0.20	0.09	0.02	0.38	.020
C3	*d*_3_	-0.32	0.09	-0.50	-0.14	< .001
*n*-back	*d*_4_	0.10	0.08	-0.04	0.24	.237
Ospan	*d*_5_	0.09	0.08	-0.06	0.24	.227
Ospan x Ambiguity	*d*_6_	-0.16	0.07	-0.28	-0.01	.026

The dummy variable *C2* was coded as 1 for the zero contingency condition, -1 for the positive condition, and 0 for the negative condition; *C3* was coded as 1 for the negative contingency condition, -1 for the positive condition, and 0 for the zero condition. The dummy variable *Ambiguity* was coded as 1 for the AU condition and -1 for the unambiguous conditions.

Ospan and *n*-back are standardized scores.

The magnitudes of causal ratings in the AU condition were not greater than those in the unambiguous condition in any of the contingency conditions, suggesting that subjects may not ignore AU observations even when they have limited cognitive resources.

Regarding the hypothesis on the relationship between WMC and ambiguity, we did not find significant interaction effects between either of the WM measures or the ambiguity on the mean of causal ratings on each level of contingency. This suggests that there is no evidence that a lower WM is associated with a greater tendency to ignore AU observations and perceive stronger causal associations between cause and effect. However, we found a significant interaction between Ospan and ambiguity on the precision of causal ratings ($Delta$ΔDIC = 7.1). The higher Ospan scores were associated with more homogeneous causal ratings in the unambiguous conditions (*d* = *d*_5_ + (-1)* *d*_6_ = 0.09 + 0.16 = 0.25). In contrast, the positive association between Ospan and the precision of causal ratings diminished in the AU condition (*d* = *d*_5_ + (1)* *d*_6_ = 0.09–0.16 = -0.07), suggesting that there was an increase in individual differences between subjects with a higher Ospan in the AU condition as compared to those in the unambiguous condition.

#### The effects of sample size

Finally, we examined the effects of sample size on the causal ratings. The causal ratings after 16 and 32 observations in the unambiguous condition were included in the analysis. Sample size did not significantly contribute to either the mean or the dispersion of the causal ratings (ΔDICs < 3). Neither *n-*back nor Ospan was associated with the mean of the causal ratings (ΔDICs < 3), but both were positively associated with the precision of the ratings (ΔDIC = 4.9 and 2.9 for contribution of *n-*back and Ospan, respectively). Neither *n-*back nor Ospan had significant interactions with sample size in the location submodel.

### Discussion

Regarding the first hypothesis, the pattern of differences between the rating results within both AU and unambiguous conditions, the results of Experiment 2 were similar to those of Experiment 1, but with substantially smaller effect sizes and thus not significant. This difference in results across the two experiments suggests that the way subjects integrated AU observations could be influenced by the level of cognitive demand required during causal reasoning. Experiment 1 indicated that subjects were more likely to treat AU observations as negative cues against causal links. However, such an evaluation requires subjects to make a decision after they have been able to appraise the causal links suggested by the unambiguous observations, after which they can then integrate their appraisals with the unambiguous observations. Experiment 2 presented subjects with causal cues sequentially, placing a substantial cognitive demand on the subjects. Subjects were left with limited resources available for the retention and integration of the AU stimuli in estimating causal strength. Instead of applying more cognitively demanding strategies, such as summarizing the previous observations, subjects might have been more likely to choose a simpler heuristic approach, such as treating each ambiguous observation as equally likely to have the effect or not. A more direct test will be presented in Experiment 3.

Regarding the second hypothesis, although there was no significant interaction between WM and ambiguity on the magnitudes of causal ratings, the association between cognitive demand and treatment of AU observations was evident in the significant interaction between Ospan and the effects of ambiguity on the variability of causal ratings. Subjects with a higher Ospan had more homogenous causal ratings (i.e., more similar to each other) in the unambiguous condition than those who had lower an Ospan. The greater homogeneity in causal ratings may be a result of similar levels of evidence retention among subjects with a higher Ospan. Meanwhile, subjects with a lower Ospan may have had a more heterogeneous evidence retention, depending on whether they selectively retained the evidence [35,36] or relied solely on the evidence presented in the most recent trials [37]. The positive association between Ospan and the homogeneity of ratings diminished in the AU condition, which might be a result of greater cognitive demand when evaluating AU observations.

We did not find a significant interaction between ambiguity and *n-*back. One possibility is that processing AU observations requires more computational processing, which was not assessed in the *n-*back test. The *n*-back test applies the single task paradigm, and does not assess divided attention, dual task processing capacity and computation, which Ospan does [20,32]. The differences in the relationship between *n*-back and Ospan also suggest that processing AU observations might be a process parallel to causal reasoning, and may involve computation.

It is also interesting that subjects’ causal ratings after 16 observations were similar to their ratings after 32 observations, suggesting that the sample size did not influence the causal strength ratings. Similar results were also observed in previous studies [38,39], where the estimates of causal strength were not sensitive to the change in observed sample sizes. The insignificant effect of sample size in combination with the results received for the AU observations in Experiments 1 and 2 suggest that the difference between causal reasoning in the AU and unambiguous conditions cannot be explained by the differences in sample size between these conditions.

## Experiment 3

Experiment 2 provided evidence that the homogeneity of subjects’ ratings with AU observations may depend on their cognitive capacities. However, the limited findings regarding the association between the two WM measures and ambiguity may be due to the limited variability of WM among college students. Experiment 3 was conducted to address the hypothesis that the choice of strategies for evaluating AU observations depends on the availability of cognitive resources that varies across different task formats. Experiment 3 used both task paradigms used in Experiments 1 and 2. The summary format of presenting evidence in Experiment 1 minimized cognitive demand (we will refer to this task format as the “low cognitive demand” format), while the trial-by-trial task in Experiment 2 required more cognitive effort to retain and online manipulate evidence (we will refer to the trial-by-trial task format as the “high cognitive demand” format).

We have discussed that one possibility for the reduced difference in the mean of the causal ratings between the AU and unambiguous conditions in Experiment 2 resulted from the increase in cognitive demand. We wished to test this hypothesis through a within-subject comparison in the current study. We hypothesized that the effects of ambiguity on causal ratings would be moderated by cognitive demand varied across the task formats. As a higher proportion of subjects would treat the AU observations as negative cues when estimating causal associations, more so under the low cognitive demand condition than the high cognitive demand condition, we hypothesized that (1) the difference between the means of the causal ratings in the AU and unambiguous conditions would be greater in the low cognitive demand condition than the high cognitive demand condition.

Based on Experiment 2, we suggested that the decline in the positive association between Ospan and the homogeneity of causal ratings in the AU condition might be due to the increased heterogeneity in evidence retention among subjects with a higher Ospan. If the homogeneity of causal ratings is associated with greater cognitive capacity, then (2) subjects’ causal ratings should be more homogenous in the low cognitive demand condition than in the high cognitive demand condition. Finally, if the presence of the AU observations can decrease similarity in evidence retention due to increased cognitive demand, (3) subjects’ causal ratings should be more homogenous in the unambiguous condition than in the AU condition.

We also examined the association between Ospan and the effects of ambiguity. If the diminished positive relationship between Ospan and the precision of the causal ratings in Experiment 2 were due to the increased cognitive demand in the AU condition, we may observe a similar result in Experiment 3. We hypothesized that (4) the association between the Ospan and the causal rating precision should be more strongly positive in the low cognitive demand condition than in the high cognitive demand condition, while the association between the Ospan and the causal rating precision should be more strongly positive in the AU condition than in the unambiguous condition.

Finally, one possible argument regarding the effects of ambiguity is that subjects might reason about the causal relations by comparing the frequencies of different observations, rather than by computing conditional probabilities. For example, the “outcome density effect” has previously been reported upon, in which causal ratings increase with the more frequent occurrence of the cause and effect [40]. Despite self-reports to the contrary, it is possible that subjects omitted the AU observations which resulted in a smaller number of both active and inactive virus samples when the cause was present than when it was absent. This, in turn, would result in a smaller magnitude of causal ratings in the AU condition. To address this, an additional condition was included in Experiment 3, wherein subjects were presented with AU observations when the cause was absent. As shown in Table 1, if subjects estimated the causal links by tallying the frequency of information, we hypothesized that (5) there would be a higher absolute causal strength rating in the AU condition than in the unambiguous condition.

### Method

#### Participants and design

A total of 126 subjects (68.3% females, mean age = 19.89, *SD* = 5.76) who were first-year psychology students at Australian National University participated in the experiment for course credit. Subjects were randomly assigned to one of the AU location conditions (*N* = 59 for AU in absence of the cause condition [AC], and *N* = 67 for AU in presence of the cause condition [PC]). Six subjects were excluded from the data analysis: three subjects pressed the wrong key during the *n*-back task, two subjects attended only one single task in Ospan, and one subject mistook the direction of the causal rating scales. The causal reasoning task included six experimental blocks comprised of two evidence types (AU vs. Unambiguous) by three contingency levels (Positive vs. Zero vs. Negative) of within-subject conditions.

#### Materials

**Causal reasoning task (High cognitive demand & Low cognitive demand):** The causal reasoning task with high cognitive demand had the same setting as the one in Experiment 2. Subjects were presented with paired stimuli sequentially. As assessing the effect of observational quantity on judgments was not the purpose of the current experiment, subjects rated causal estimates only after observing all 32 paired stimuli instead of rating them after only 16 observations. The causal reasoning task with the low cognitive demand applied the paradigm used in Experiment 1, in which subjects observed all evidence in one experimental block on a single screen. Subjects observed the evidence and retained the information until the next page, on which they rated the causal relations between the cause and the effect. The AU effect stimuli were presented in cases where the chemical was absent for the AU in absence of the cause condition (AC), as well as where the chemical was present for the AU in presence of the cause condition (PC).

***N*-back and Ospan tasks:** Experiment 2 revealed that the section of 1- to 3-back set sizes for that select sample resulted in skewed test results, indicating that the norm of the *n*-back test scores of the university students was higher than the general norm reported in previous research which used more general test populations. To avoid this ceiling effect, Experiment 3 selected 2-back and 3-back trials only, each of which was repeated for four trials. To reduce cognitive fatigue we reduced the total number of trials in the Ospan task to two trials for each set size, resulting in a total Ospan score of 50 instead of 75.

### Results

#### Effects of cognitive demand related to task formats

**Fig 4:**
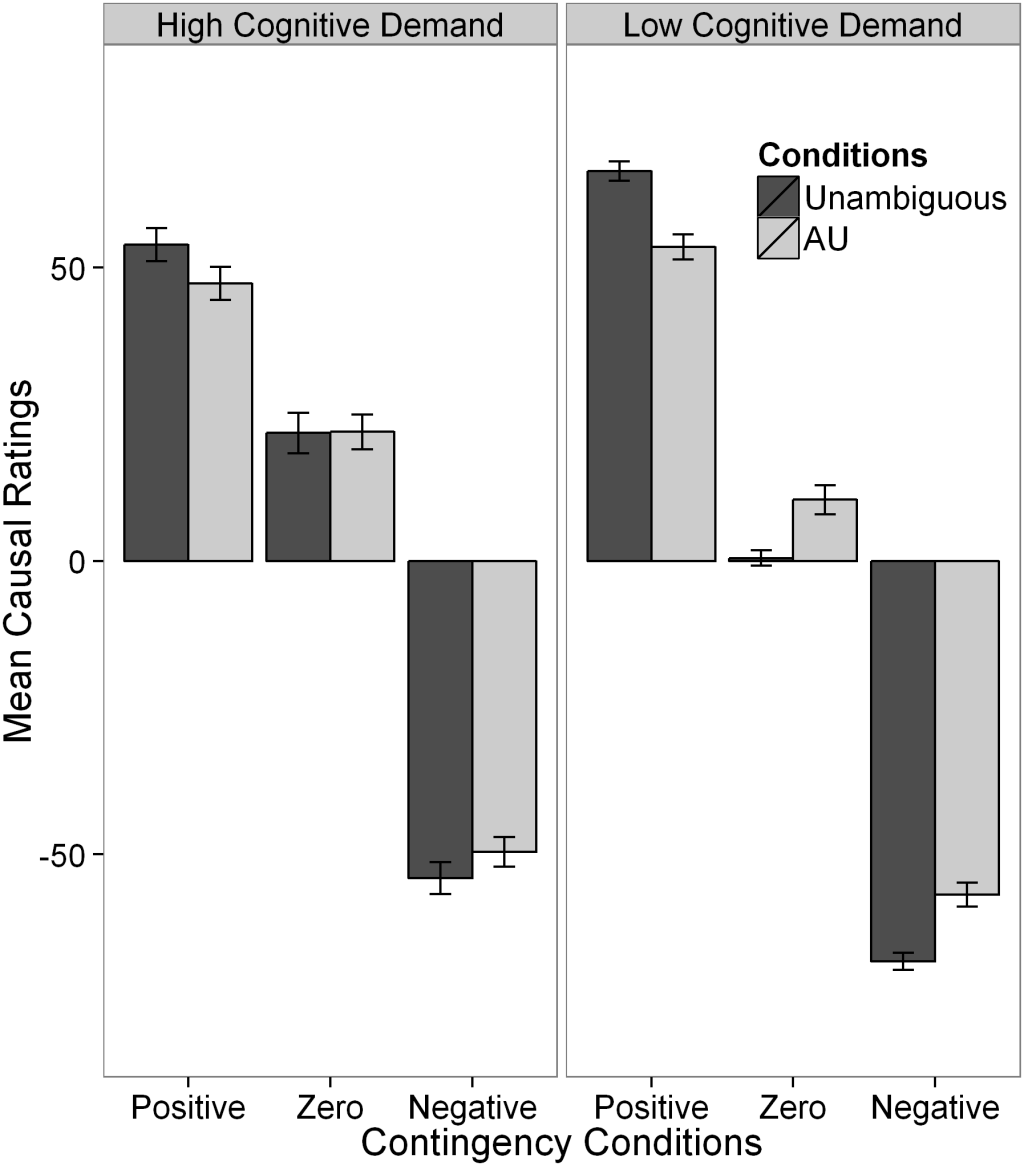
Mean causal ratings across conditions in Experiment 3. Error bars are standard errors.

Fig 4 summarizes the mean causal ratings and confidence ratings for the different conditions. Beta GLMs were first applied to model the effects of cognitive demand related to task format, contingency, and ambiguity on causal ratings. The final model was based on 119 subjects, as two influential cases were deleted through cross-validation via case-wise deletion. Cognitive demand had significant main effects on the causal ratings in both the mean and the precision submodels (ΔDIC = 18.91 and 15.11), while ambiguity did not have significant main effects in either submodel (ΔDICs < 3). However, there was a significant interaction between ambiguity and cognitive demand on the mean of the causal ratings ($Delta$ΔDIC = 16.58). This interaction effect was also significantly moderated by the contingency levels ($Delta$ΔDIC = 19.28 for the three-way interaction effect). To disentangle the complex interaction effects, we assessed the effects of ambiguity and cognitive demand for each of the contingency levels.

**Positive contingency condition:** Table 5 shows the results of the best model for the causal ratings of the positive contingency condition. Both cognitive demand and ambiguity had substantial effects on the mean of the causal ratings in the positive contingency condition ($Delta$ΔDIC = 11.53 and 11.51, respectively). The ratings of the high cognitive demand condition were significantly lower than those of the low condition (*$d$b* = -0.13, $95%CI = [0.02, 0.49]$95%*CI =* [-0.20, -0.07]), while the ratings in the AU condition were also significantly lower than those of the unambiguous condition (*$d$b* = -0.13, $95%CI = [0.02, 0.49]$95%*CI =* [-0.20, -0.06]). There was an interaction between ambiguity and cognitive demand ($Delta$ΔDIC = 5.52, *b* = 0.07, $95%CI = [0.02, 0.49]$95%*CI =* [0.01, 0.13]). In line with our first hypothesis, the magnitude of the effect of ambiguity in the high cognitive demand condition was significantly smaller than in the low cognitive demand condition ($b$*b* = -0.06 for effects of ambiguity in the high load and $b$*b* = -0.20 for effects of ambiguity in the low load).

**Table 5:** Effects of Ambiguity on Causal Ratings of the Positive Contingency in Experiment 3.

Variables	Coefficient	*SE*	2.5%CI	97.5%CI	*p*
Random Intercept	0.96	0.11	0.71	1.19	
**Location Submodel**					
Intercept	1.30	0.05	1.20	1.39	< .001
Ambiguity	-0.13	0.03	-0.19	-0.07	.001
Cognitive Demand	-0.12	0.03	-0.18	-0.06	< .001
Ambiguity x Cognitive Demand	0.07	0.03	0.01	0.13	.003
**Precision Submodel**					
Intercept	2.51	0.07	2.36	2.55	< .001
Ambiguity	-0.07	0.06	-0.20	0.05	.764
Cognitive Demand	-0.31	0.08	-0.47	-0.16	< .001

*Ambiguity* was coded as 1 for the AU and -1 for the unambiguous conditions. *Cognitive demand* was coded as 1 for the high cognitive demand and -1 for the low cognitive demand conditions.

**Negative contingency condition:** Table 6 shows the results of the best model for the causal ratings in the negative contingency condition. Both ambiguity and cognitive demand had substantial effects on the mean of the causal ratings ($Delta$ΔDIC = 33.45 and 26.91). The ratings in the AU condition were significantly more positive than those in the unambiguous condition (*$d$b* = 0.22, $95%CI = [0.02, 0.49]$95%*CI =* [0.15, 0.28]), while the ratings in the high cognitive demand condition were significantly more positive than those in the low cognitive demand condition (*$d$b* = 0.19, $95%CI = [0.02, 0.49]$95%*CI* = [0.12, 0.26]). There was also a significant interaction between ambiguity and cognitive demand ($Delta$ΔDIC = 4, *b* = -0.09, $95%CI = [0.02, 0.49]$95%*CI* = [-0.15, -0.02]). Again, in line with the first hypothesis, the increase in the AU condition in the high cognitive demand condition was smaller than in the low cognitive demand condition ($b$*b* = 0.13 for effects of ambiguity under the high load and $b$*b* = 0.31 for effects of ambiguity under the low load).

**Table 6:** Effects of Ambiguity in Presence of the Cause on Causal Ratings of the Negative Contingency in Experiment 3.

Variables	Coefficient	*SE*	2.5%CI	97.5%CI	*p*
Random Intercept	1.04	0.13	0.81	1.34	
**Location Submodel**					
Intercept	-1.34	0.05	-1.44	-1.17	< .001
Ambiguity	0.12	0.03	0.11	0.28	< .001
Cognitive Demand	0.17	0.03	0.11	0.26	< .001
Ambiguity x Cognitive Demand	-0.08	0.03	-0.15	-0.02	.023
**Precision Submodel**					
Intercept	2.54	0.07	2.11	2.68	< .001
Ambiguity	-0.10	0.07	-0.25	0.04	.159
Cognitive Demand	-0.30	0.08	-0.45	-0.14	< .001

*Ambiguity* was coded as 1 for the AU and -1 for the unambiguous condition. *Cognitive Demand* was coded as 1 for the high cognitive demand and -1 for the low conditions.

Cognitive demand also had a substantial effect on the variability of causal ratings ($Delta$ΔDIC = 9, respectively). The ratings were more heterogeneous in the high cognitive demand condition than they were in the low cognitive demand condition ($Delta$*d* = -0.30, $95%CI = [0.02, 0.49]$95%*CI* = [-0.45, -0.14]).

**Zero contingency condition:** Because there were a substantial number of zero values in the zero contingency condition, we applied a mixture model to model the zero and non-zero components simultaneously. The mixture model first recruits the binomial distribution to assess the likelihood of a case to be in the zero versus non-zero component. It then applies the beta distribution to model the non-zero component. The results of the final model are shown in Table 7. Results showed that both ambiguity and cognitive demand made substantial contributions to the tendency of subjects to rate zero in the zero contingency condition ($Delta$ΔDIC = 25.28 and 92.65, respectively). The ratings were significantly less likely to be zero in the AU condition than they were in the unambiguous condition ($theta$θ = -0.56, 95%*CI =* [-0.78, -0.66], $95%CI = [-0.94, -0.25]$odds ratio = 0.57), and also significantly less likely to be zero in the high cognitive demand conditions than in the low cognitive demand condition ($theta$θ = -1.05, 95%*CI =* [-1.29, -0.81], $95%CI = [-1.57, -0.86]$odds ratio = 0.35).

**Table 7:** Effects of Ambiguity on Causal Ratings of the Zero Contingency in Experiment 3.

Variables	Coefficient	*SE*	2.5%CI	97.5%CI	*p*
**Non-Zero Location Submodel**					
Intercept	0.48	0.05	0.38	0.58	< .001
Cognitive Demand	0.12	0.05	0.01	0.22	.040
**Non-Zero Precision Submodel**					
Intercept	1.72	0.09	1.49	1.88	< .001
Ambiguity	0.20	0.08	0.05	0.37	.015
**Zero Component**					
Intercept	-0.07	0.12	-0.32	0.14	.600
Ambiguity	-0.56	0.11	-0.78	-0.33	< .001
Cognitive Demand	-1.05	0.12	-1.29	-0.81	< .001
Ambiguity x Cognitive Demand	0.61	0.12	0.37	0.83	< .001

*Ambiguity* was coded as 1 for the AU and -1 for the unambiguous conditions. *Cognitive Demand* was coded as 1 for the high cognitive demand and -1 for the low cognitive demand conditions.

There was a significant interaction between cognitive demand and ambiguity (ΔDIC = 27.74, $theta$θ = 0.61, 95%*CI =* [0.37, 0.86]), as the ratings were similarly likely to be zero in both the AU and unambiguous conditions in the high cognitive demand condition ($theta$θ = 0.05 for effects of ambiguity under the high load, and $theta$θ = -1.17 for effects of ambiguity under the low load). Neither of the two WM measures nor their interactions with ambiguity or cognitive demand made significant contributions to the model (ΔDICs < 3). Turning to the non-zero component, cognitive demand affected the mean of the causal ratings, whereby the ratings in the non-zero component were substantially higher in the high cognitive demand condition than they were in the low cognitive demand condition.

**Interim summary:** Hypothesis 1, that the effects of ambiguity depend on the availability of cognitive resources, was supported, as a significant interaction between ambiguity and cognitive demand was found across all contingency conditions. The difference in the mean causal ratings between the AU condition and the unambiguous AU condition was greater in the low cognitive demand condition than in the high cognitive demand condition. Hypothesis 2 was supported by the significant effect of cognitive demand on the homogeneity of causal ratings. Subjects had substantially more homogenous causal ratings in the low cognitive demand condition than they did in the high cognitive demand condition. Hypothesis 3 was partially supported, whereby subjects’ causal ratings in the AU condition were significantly more heterogeneous than they were in the unambiguous condition in the negative contingency condition. The other two hypotheses are examined in the following section.

#### Effects of internal capacity

To test Hypothesis 4, beta GLMs were applied to examine the interaction effects between ambiguity, cognitive demand, and the two WM measures on causal ratings of all three contingency conditions. The best model selected via DIC values is shown in Table 8. Higher Ospan was associated with greater homogeneity in causal ratings (*d*_5_ = 0.17). In support of the fourth hypothesis, there was a significant interaction between Ospan and cognitive demand in the precision submodel (ΔDIC = 16.0, *d*_6_ = -0.14, 95%CI = [-0.21, -0.08]). Higher Ospan was associated with lower variability in the low cognitive demand condition (*d* = *d*_5_ *+* (-1) * *d*_6_ = 0.17 + (-1) * (-0.14) = 0.31). The positive association diminished in the high cognitive demand condition (*d* = *d*_5_ *+* (1) * *d*_7_ = 0.17 + (1) * (-0.14) = 0.03). However, we did not find a significant interaction between Ospan and ambiguity (ΔDIC < 3).

**Table 8:** Beta Regression on Causal Ratings Predicted by Experimental Factors and WMC.

Variables	Parameter	Coefficient	*SE*	2.5%CI	97.5%CI	*p*
**Location Submodel**						
Intercept	*b*_0_	0.06	0.02	0.02	0.09	.014
C2	*b*_1_	0.18	0.03	0.15	0.24	< .001
C3	*b*_2_	-1.40	0.03	-1.46	-1.36	< .001
Cognitive Demand	*b*_3_	0.09	0.02	0.05	0.10	< .001
Ambiguity	*b*_4_	0.02	0.02	-0.02	0.05	.081
**Precision Submodel**						
Intercept	*d*_0_	2.24	0.04	2.16	2.31	< .001
C2	*d*_1_	0.07	0.05	0.08	0.10	.002
C3	*d*_2_	-0.05	0.05	-0.22	-0.02	.939
Ambiguity	*d*_3_	-0.00	0.04	-0.07	0.07	.824
Cognitive Demand	*d*_4_	-0.37	0.04	-0.31	-0.20	< .001
Ospan^a^	*d*_5_	0.17	0.04	0.10	0.24	.001
Ospan x Cognitive Demand	*d*_6_	-0.14	0.04	-0.22	-0.08	< .001

The dummy variable *C2* was coded 1 for the zero contingency condition, -1 for the positive condition and 0 for the negative condition. *C3* was coded 1 for the negative contingency condition, -1 for the positive condition and 0 for the zero condition. *Ambiguity* was coded 1 for the AU condition and -1 for the unambiguous conditions. *Cognitive Demand* was coded 1 for the high cognitive demand condition and -1 for the low conditions.

^a^Ospan is standardized.

#### Effects of AU stimuli location

A separate model was constructed to examine the effects of the location of AU information on the causal ratings in the AU condition. The distribution of the AU information did not have significant main effects on either the mean or the precision of causal ratings (ΔDICs < 3). However, there was a significant interaction between contingency and the distribution of ambiguity (ΔDIC = 12.03). The post hoc repeated comparison suggests that subjects in the AC condition had substantially more positive causal ratings than those in the PC condition for the positive contingency condition (*b* = 0.17, 95%CI = [0.07, 0.27]), and substantially more negative causal ratings than those in the PC condition for the negative contingency condition (*b* = -0.11, 95%CI = [-0.21, -0.01]). There was no significant difference between the two conditions in the ratings of the zero contingency conditions (*b* = 0.06, 95%CI = [-0.03, 0.13]). No other significant interactions were found.

### Discussion

Ambiguity had significant main effects on the mean of the causal ratings in each of the contingency conditions. Consistent with the results of Experiment 1, the direction of the effects for both positive and negative contingency conditions were opposite to those predicted by the assumption that subjects omitted the AU observations. In support of the first hypothesis, the effects of ambiguity depended on the cognitive demand involved in the task. The difference between the AU condition and the unambiguous condition was greater under the low cognitive demand condition (summary format task paradigm) than under the high cognitive demand condition (trial-by-trial task paradigm). In addition, the results of the precision submodel suggest that the precision of the causal ratings may be associated with the homogeneity of evidence retention (Hypothesis 2), and that the presence of AU observations decreases such homogeneity (Hypothesis 3). Evaluating AU information, possibly a deliberative process, requires extra cognitive resources in addition to those used to evaluate causal relations. Subjects may process AU information adaptively depending on their environment [41]. When time and cognitive resources were restricted, causal reasoning with AU observations were compromised by the adoption of more heuristic strategies, such as treating each AU observation as equally likely for the effect to be present or absent.

With regard to Hypothesis 4, we found that higher Ospan is associated with higher homogeneity of causal ratings in the low cognitive demand condition. The relationship diminished in the high cognitive demand condition. In the low cognitive demand condition, subjects with higher Ospan may have had the advantage of being able to retain more evidence while reasoning, with similarity in evidence retention accounting for the homogeneity in causal ratings. In the high cognitive demand condition, the demand on memory and the online computation may have exceeded the limit of the type of WM capacity assessed by Ospan (e.g., given the maximum set size was seven). If so, then subjects with higher Ospan did not have a greater advantage in evaluating the evidence at the end of each trial over those who had lower Ospan. The interaction between Ospan and ambiguity conditions also diminished, and was not observed in the high cognitive demand condition. It is possible that the cognitive demand in Experiment 3 was higher than in Experiment 2 due to the removal of the stop-point after the initial 16 observations. Subjects with higher Ospan did not have an advantage in evaluating observations in unambiguous conditions, and they became insensitive to the increased cognitive demand demanded by the AU condition.

Finally, presenting AU observations, regardless of whether the cause was present or absent, reduced causal strength estimates when the causal directions were unambiguous resulted in a tendency for positive ratings when the causal direction was unknown (i.e., the zero contingency condition). This suggests that the results in Experiment 2 were not due to subjects judging causal strength by relying on the frequency of the observations in which the effect was present. However, we did find effects of the distribution of AU observations on causal ratings. Subjects perceived the causal strength between the candidate cause and the effect as being stronger when the AU observations were presented in absence of the cause. One possible explanation for this is that the effect status in absence of the cause provided indications of the causal strength between the background causes (the causes other than the candidate cause). The AU observations as negative cues reduced the estimated causal strength between the background causes and effects. This further resulted in an increase in the estimates of the causal strength of the candidate cause, given that the probability of the effect in the presence of the candidate cause was unchanged.

## General Discussion

The studies reported here investigated the mechanisms of causal reasoning with ambiguous information due to partially-known observations. First, all experiments demonstrated that people do not simply ignore ambiguous-unknown (AU) observations in causal reasoning. Experiments 1 and 3 demonstrated that the effects of ambiguity on causal strength ratings differed systematically from the effects predicted by assuming that people ignore AU evidence. Our results suggest that the findings of Marsh and Ahn [11], that subjects might exclude AU evidence when they do not have the frequency estimates of unknown stimuli. Subjects did not imply that they excluded these stimuli when reasoning about causal associations. Observations with incomplete information (i.e., where only the status of one variable is known) are sources of ambiguity, and may influence estimates of probabilities [42,43] that are used for estimating the contingency between cause and effect.

Second, we explored how limitations on cognitive resources could influence processing and integrating AU information. We explored the impacts of external cognitive demand and internal cognitive capacities on subjects’ behaviors under ambiguity. Both Experiment 2 and Experiment 3 demonstrated that the effects of ambiguity on causal ratings diminishes substantially when external cognitive demand is high. As suggested in the previous study, processing ambiguity may involve evaluating probabilities related to each possible outcome as well as arithmetic operations [8]. The current results imply that causal reasoning with AU information could involve deliberative processes, including the evaluation of available evidence, estimating AU information and integrating observations in updating beliefs about causal strength.

Regarding the relationships between the two WM measures and ambiguity, we found there to be a significant interaction between Ospan and ambiguity on the variability in causal ratings in the AU condition in Experiment 2. It is possible that subjects with higher Ospan have the capacity to enable the process of dealing with AU observations. Greater variability in ratings may indicate a greater variability in strategy choice.

### Strategies for treating AU observations

This paper presented an investigation into the strategies subjects may adopt in processing AU observations. In general, when the direction of the causation was unambiguous (i.e., in the positive and negative contingency conditions), subjects’ ratings regarding the strength of the causal associations diminished when there was AU information. Garcia-Retamero and Rieskamp [2] used a forced two-alternative-choice task and found that subjects were more likely to treat missing cues as negative cues when these missing cues were distributed unequally across two alternative choices. In our three experiments, the unknown observations were not equally distributed across the four types of covariation observations; (i.e., they were all distributed in the presence of the cause, or in the absence of the cause in Experiment 3). It is possible that subjects who treat AU observations as evidence are less likely to endorse the hypothesis that the cause has an effect. This in turn would result in a weaker estimation of causal strength than if the AU observations were seen as equally likely to have the effect present or absent. In the AU zero contingency condition the deviance of causal ratings from zero, again, suggested that subjects might not treat the AU observations as symmetrically distributed. That is, they did not perceive the AU viruses as equally likely to be active or inactive. The general positive ratings suggest subjects were more likely to perceive a positive association when the direction of the causation was unknown.

However, it is unclear whether the general tendency to perceive a positive causal association is due to one’s prior beliefs or to the nature of the evidence. A recent study conducted by Johnson and Keil [44] suggested that causal reasoning under ambiguous situations is modulated by the base rate of the effects in evidence, i.e., the proportion of the effect-present events out of the total observations. The current three experiments presented a high base rate of effect in the zero contingency condition, and it is possible that subjects dealt with the ambiguity in causal direction relying on this base rate.

Furthermore, we found that the distribution of AU information had significant effects on subjects’ causal ratings under the AU condition. The inequality between the probabilities when the cause was absent versus present resulted in a perceived positive contingency between the cause and the effect. The results show that participants were sensitive to how missing information was distributed in an environment, and might have frequently selected a strategy responsively. However, the means of the causal ratings for the zero contingency condition were similar in the AU and unambiguous conditions when cognitive demand was high, suggesting that subjects may have been more likely to adopt the symmetric imputation strategy when cognitive resources were restricted.

### Future Directions

Several questions remain to be addressed in this line of research. First, it is unclear whether WM functions, other than simultaneous storage and processing, are contributing to processing ambiguous observations. Recent theories of WM regard it as a multifaceted construct [20,21,22], which comprise of a range of domain-generic executive functions. The unique WM functions involved in Ospan independent from *n-*back also include divided attention. The role of divided attention control in evaluating ambiguous stimuli may inform the relationship between ambiguity processing and causal reasoning. An association between divided attention and causal reasoning under ambiguity suggests that processing ambiguous observations can occur parallel to the process of causal reasoning. Future studies may include tasks that separately measure divided attention (e.g., reading span task) and computation [21].

It is also still unclear whether the choice of strategy in processing ambiguous observations is associated with WMC or cognitive resources. Studies in developmental psychology suggest that the strategies selected by children when assessing ambiguous observations may depend on the extent that the children acknowledge the possible states or alternative hypotheses of the observations [45,46]. The capacity for holding and evaluating multiple hypotheses, therefore, can also influence strategy selection. If the selection of a strategy depends on WMC, this may be due to strategies differing in their cognitive demands. Subjects may adaptively choose a strategy depending on the available cognitive resources. On the other hand, if the strategy is independent of cognitive capacity, the treatment of ambiguous observations may be a result of individual differences such as ambiguity aversion or seeking.

The higher dispersion of causal ratings among subjects with higher Ospan may also be due to factors relating to individual differences other than cognitive processes. Pushkarskaya et al. [8] observed that the activation of the inferior parietal lobe is moderated by individual differences in the tolerance of ambiguity. Pushkarskaya et al. [8] found that subjects who were more ambiguity averse had a greater activation in the face of ambiguity than those who were less ambiguity averse. They suggested that subjects who were more ambiguity averse were more likely to employ deliberative processing when presented with ambiguous stimuli. Future study may integrate individual difference assessments such as the ambiguity-probability tradeoff task [47] and ambiguity tolerance scale [48] to better understand the mechanisms of ambiguity processing in causal reasoning.

There is also some evidence that the format of presentation of causal evidence may influence the underlying causal inferences, and that the summary format may enhance the detection of contingency in data [49]. It is possible that the way in which subjects process ambiguous information may also be influenced by the task formats in addition to the cognitive demand. Future studies may manipulate cognitive load by adding a second task to the summary format to better understand how cognitive demand can influence ambiguity processing.

Finally, we note that in this paper we have investigated only one way of inducing ambiguity: through the absence of information. Ambiguity has been operationalized in a variety of ways throughout psychology, ranging from bi-stable visual stimuli to probability intervals. Likewise, there are several ways of presenting causal evidence, including verbal descriptions as well as numeric information about frequencies or conditional probabilities [49]. Future studies may investigate alternative methods for inducing ambiguity in cause-effect information, such as through multifarious descriptions or vague probabilities, to extend our understanding of causal reasoning under ambiguity.

## Supporting Information

S1 Appendix Example Experimental Materials. (PDF)

S2 Appendix Introduction of Beta GLM and Model Syntax. (PDF)

S1 Dataset Experiment Data. (XLSX)

## References

[1] Smithson MJ, Bartos T, Takemura K. Human judgment under sample space ignorance Risk Decis Policy. Cambridge University Press; 2000;5: 135–150. Available: http://journals.cambridge.org/abstract_S1357530900000144

[2] Garcia-Retamero R, Rieskamp J. Do people treat missing information adaptively when making inferences? Q J Exp Psychol. 2009;62: 1991–2013. 10.1080/17470210802602615 19241222

[3] Griffiths TL, Tenenbaum JB. From mere coincidences to meaningful discoveries. Cognition. 2007;103: 180–226. 10.1016/j.cognition.2006.03.004 16678145

[4] Griffiths TL, Tenenbaum JB. Theory-based causal induction. Psychol Rev. United States: American Psychological Association; 2009;116: 661–716. 10.1037/a0017201 19839681

[5] Griffiths TL, Sobel DM, Tenenbaum JB, Gopnik A. Bayes and Blickets: Effects of Knowledge on Causal Induction in Children and Adults. Cogn Sci. Blackwell Publishing Ltd; 2011;35: 1407–1455. 10.1111/j.1551-6709.2011.01203.x PMC3208735 21972897

[6] Sobel DM, Munro SE. Domain generality and specificity in children’s causal inference about ambiguous data. Dev Psychol. 2009;45: 511–524. 10.1037/a0014944 19271835

[7] Griffiths TL, Tenenbaum JB. Structure and strength in causal induction. Cogn Psychol. United States: Elsevier Inc; 2005;51: 334–384. 10.1016/j.cogpsych.2005.05.004 16168981

[8] Pushkarskaya H, Liu X, Smithson M, Joseph JE. Beyond risk and ambiguity: deciding under ignorance. Cogn Affect Behav Neurosci. 2010;10: 382–391. 10.3758/CABN.10.3.382 20805539

[9] Smithson M. Conflict Aversion: Preference for Ambiguity vs Conflict in Sources and Evidence. Organ Behav Hum Decis Process. 1999;79: 179–198. 10.1006/obhd.1999.2844 10471360

[10] Black M. Vagueness. An Exercise in Logical Analysis. Philosophy of Science. 1937 p. 427 10.1086/286476

[11] Marsh JK, Ahn W-K. Spontaneous assimilation of continuous values and temporal information in causal induction. J Exp Psychol Learn Mem Cogn. 2009;35: 334–52. 10.1037/a0014929 19271850 PMC2826811

[12] Buehner MJ, Cheng PW, Clifford D. From Covariation to Causation: A Test of the Assumption of Causal Power. J Exp Psychol Learn Mem Cogn. United States: American Psychological Association; 2003;29: 1119–1140. 10.1037/0278-7393.29.6.1119 14622051

[13] Fox CR, Rottenstreich Y. Partition Priming in Judgment Under Uncertainty. Psychol Sci. SAGE Publications; 2003;14: 195–200. 10.1111/1467-9280.02431 12741740

[14] Del Missier F, Mäntylä T, Hansson P, Bruine de Bruin W, Parker AM, Nilsson L-G. The multifold relationship between memory and decision making: An individual-differences study. J Exp Psychol Learn Mem Cogn. 2013;39: 1344–64. 10.1037/a0032379 23565790 PMC4160880

[15] Mutter SA, Pliske RM. Judging Event Covariation: Effects of Age and Memory Demand. Journals Gerontol Ser B Psychol Sci Soc Sci. 1996;51B: P70–P80. 10.1093/geronb/51B.2.P70 8785689

[16] Satpute AB, Fenker DB, Waldmann MR, Tabibnia G, Holyoak KJ, Lieberman MD. An fMRI study of causal judgments. Eur J Neurosci. 2005;22: 1233–8. 10.1111/j.1460-9568.2005.04292.x 16176366

[17] Buehner M, Krumm S, Pick M. Reasoning = working memory≠attention. Intelligence. 2005;33: 251–272.

[18] Jaeggi SM, Buschkuehl M, Perrig WJ, Meier B. The concurrent validity of the N-back task as a working memory measure. Memory. Routledge; 2010;18: 394–412. 10.1080/09658211003702171 20408039

[19] Lange ND, Thomas RP, Buttaccio DR, Illingworth DA, Davelaar EJ. Working memory dynamics bias the generation of beliefs: the influence of data presentation rate on hypothesis generation. Psychon Bull Rev. 2013;20: 171–6. 10.3758/s13423-012-0316-9 23055141

[20] Conway ARA, Kane MJ, Bunting MF, Hambrick DZ, Wilhelm O, Engle RW. Working memory span tasks: A methodological review and user’s guide. Psychon Bull Rev. 2005;12: 769–786. 16523997 10.3758/bf03196772

[21] Oberauer K, Süß H-M, Wilhelm O, Wittman WW. The multiple faces of working memory. Intelligence. 2003;31: 167–193. 10.1016/S0160-2896(02)00115-0

[22] Unsworth N, Spillers GJ. Working memory capacity: Attention control, secondary memory, or both? A direct test of the dual-component model. J Mem Lang. 2010;62: 392–406.

[23] Jenkins HM, Ward WC. Judgment of contingency between responses and outcomes. Psychol Monogr Gen Appl. 1965;79: 1. 10.1037/h0093874 14300511

[24] Cheng PW. From Covariation to Causation: A Causal Power Theory. Psychol Rev. Washington: American Psychological Association; 1997;104: 367–405.

[25] Shou Y, Smithson M. Effects of question formats on causal judgments and model evaluation. Front Psychol. Frontiers; 2015;06 10.3389/fpsyg.2015.00467 PMC4404718 25954225

[26] Smithson M, Merkle EC. Generalized Linear Models for Categorical and Continuous Limited Dependent Variables. Boca Raton, FL: Chapman and Hall/CRC Press; 2013.

[27] Spiegelhalter DJ, Best NG, Carlin BP, van der Linde A. Bayesian measures of model complexity and fit. J R Stat Soc Ser B Statistical Methodol. 2002;64: 583–639. 10.1111/1467-9868.00353

[28] Liu H-H, Colman AM. Ambiguity aversion in the long run: Repeated decisions under risk and uncertainty. J Econ Psychol. 2009;30: 277–284. 10.1016/j.joep.2009.02.001

[29] Garcia-Retamero R, Rieskamp J. Adaptive mechanisms for treating missing information: A simulation study. Psychol Rec. 2008;58: 547–568.

[30] Chuderski A, Necka E. The contribution of working memory to fluid reasoning: Capacity, control, or both? J Exp Psychol Learn Mem Cogn. 2012;38: 1689–1710. 10.1037/a0028465 22612170

[31] Jaeggi SM, Studer-Luethi B, Buschkuehl M, Su Y-F, Jonides J, Perrig WJ. The relationship between n-back performance and matrix reasoning implications for training and transfer. Intelligence. 2010;38: 625–635.

[32] Faraco CC, Unsworth N, Langley J, Terry D, Li K, Zhang D, et al Complex span tasks and hippocampal recruitment during working memory. Neuroimage. 2011;55: 773–787. 10.1016/j.neuroimage.2010.12.033 21182968

[33] Owen AM, McMillan KM, Laird AR, Bullmore E. N-back working memory paradigm: a meta-analysis of normative functional neuroimaging studies. Hum Brain Mapp. 2005;25: 46–59. 10.1002/hbm.20131 15846822 PMC6871745

[34] Unsworth N, Heitz RP, Schrock JC, Engle RW. An automated version of the operation span task. Behav Res Methods. 2005;37: 498–505. 10.3758/BF03192720 16405146

[35] Mutter SA, Williams TW. Aging and the detection of contingency in causal learning. Psychol Aging. 2004;19: 13–26. 15065928 10.1037/0882-7974.19.1.13

[36] Mutter SA, Strain LM, Plumlee LF. The role of age and prior beliefs in contingency judgment. Mem Cognit. 2007;35: 875–884. 10.3758/BF03193462 17910173

[37] Sasaki T. Individual differences in working memory capacity in recency effects: from the recall process. Psychol Rep. 2009;104: 545–8. 19610484 10.2466/PR0.104.2.545-548

[38] Liljeholm M, Cheng PW. The influence of virtual sample size on confidence and causal-strength judgments. J Exp Psychol Learn Mem Cogn. 2009;35: 157–172. 10.1037/a0013972 19210088

[39] Lu H, Yuille AL, Liljeholm M, Cheng PW, Holyoak KJ. Bayesian generic priors for causal learning. Psychol Rev. United States: American Psychological Association; 2008;115: 955–984. 10.1037/a0013256 18954210

[40] Perales JC, Shanks DR. Models of covariation-based causal judgment: a review and synthesis. Psychon Bull Rev. United States: Springer-Verlag; 2007;14: 577–596. 10.3758/bf03196807 17972719

[41] Garcia-Retamero R, Müller SM, Catena A, Maldonado A. The power of causal beliefs and conflicting evidence on causal judgments and decision making. Learn Motiv. Elsevier Inc; 2009;40: 284–297. 10.1016/j.lmot.2009.04.001

[42] Baron J. Thinking and deciding. New York: Cambridge University Press; 2008.

[43] Camerer C, Weber M. Recent developments in modeling preferences: Uncertainty and ambiguity. J Risk Uncertain. Springer Netherlands; 1992;5: 325–370. 10.1007/bf00122575

[44] Johnson SGB, Keil FC. Causal inference and the hierarchical structure of experience. J Exp Psychol Gen. 2014;143: 2223–2241. 10.1037/a0038192 25347533 PMC4244254

[45] Beck SR, Robinson EJ, Carroll DJ, Apperly IA. Children’s thinking about counterfactuals and future hypotheticals as possibilities. Child Dev. 2006;77: 413–426. 16611181 10.1111/j.1467-8624.2006.00879.x

[46] Thomas RP, Dougherty MR, Sprenger AM, Harbison JI. Diagnostic hypothesis generation and human judgment. Psychol Rev. 2008;115: 155–185. 10.1037/0033-295X.115.1.155 18211189

[47] Lauriola M, Levin IP. Relating individual differences in Attitude toward Ambiguity to risky choices. J Behav Decis Mak. 2001;14: 107–122. 10.1002/bdm.368

[48] MacDonald AP. Revised scale for ambiguity tolerance: Reliability and validity. Psychol Rep. Ammons Scientific; 1970;26: 791–798. 10.2466/pr0.1970.26.3.791

[49] Vallée-Tourangeau F, Payton T, Murphy RA. The impact of presentation format on causal inferences. Eur J Cogn Psychol. Routledge; 2008;20: 177–194. 10.1080/09541440601056588

