# Supplementary material for: Adapting to an Uncertain World: Cognitive Capacity and Causal Reasoning with Ambiguous Observations
Source: PLoS One. 2015 Oct 15;10(10):e0140608. doi: 10.1371/journal.pone.0140608 (PMC4607167; doi:10.1371/journal.pone.0140608)
Supplement: S1 Appendix — (PDF) [file pone.0140608.s001.pdf]

S1 Appendix. Experimental Materials

Experiment 1 Causal Reasoning Task

1. **Instruction**

"Suppose you are a researcher at a Neurovirology Research Institute. Your team receives a project that investigates the effects of a list of chemicals on viruses that causes neurological diseases. The chemicals on the list are all from different molecular classifications. You want to test whether a certain chemical activates or inactivates a certain type of virus.

You will observe the results from a number of experiments. In each of these experiments, a testing chemical is selected from the list and is tested on a number of virus samples of the same type. You will be shown the status (activated or inactivated) of sample viruses, and will be told whether each sample virus has been or has not been exposed to the chemical. For each experiment, after observing the results, your task is to evaluate the influence of the chemical on the virus."

2. Example Instruction and Measurement Questions

CHEMICAL ID: XXX23

This legend tells you how the result of each sample is described:
The **Blue virus** picture indicates the virus is **inactive**,
The **Red virus** picture indicates the virus is **active**.

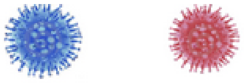

Inactivated Activated

Then, you will see results of two sets of virus samples.
The first set of virus samples have not been exposed to the testing chemical

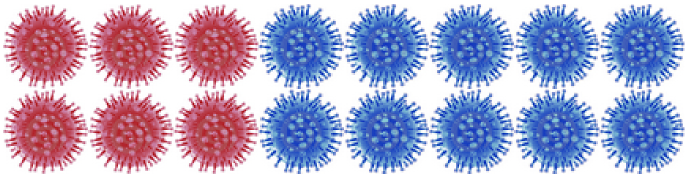

The second set of virus samples, on the other hand, have been exposed to the testing chemical

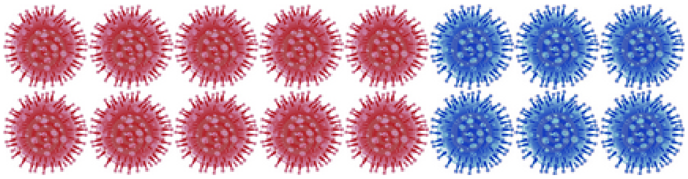

The question below asks you to estimate the relationship between the testing chemical and this type of virus.
How likely do you think it is that this type of chemical activates or inactivates this type of virus?
You rate your estimate on the scale from -100 to 100.

A ***negative*** rating (value smaller than 0) means the chemical inactivates this type of virus;
A ***positive*** rating (value greater than 0) means the chemical activates this type of virus;
A ***zero*** rating is appropriate when you think the chemical has no effects on the activation of this type of virus.

Note: A chemical cannot have both inactivating and activating effects on the viruses at the same time!

	Definitely Inactivates This Virus Type				Has No Effects on This Virus Type				Definitely Activates This Virus Type
	-100	-75	-50	-25	0	25	50	75	100
This Chemical									

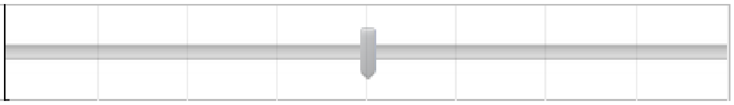

3. **Example stimuli:**

Unambiguous positive contingency condition

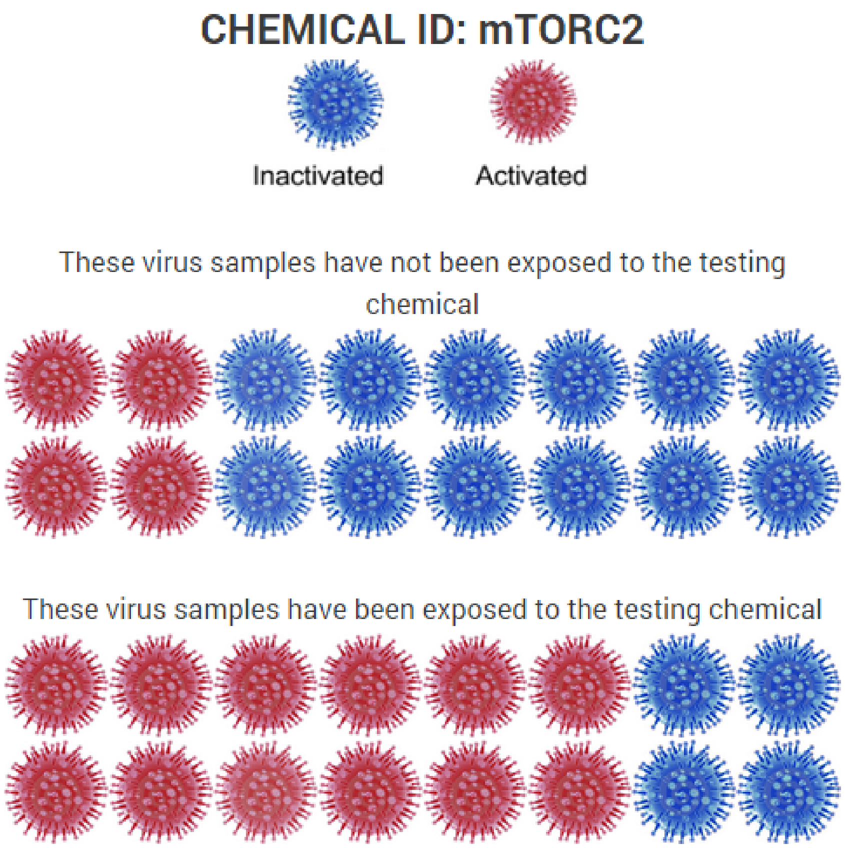

Ambiguous-Unknown Positive Contingency Condition

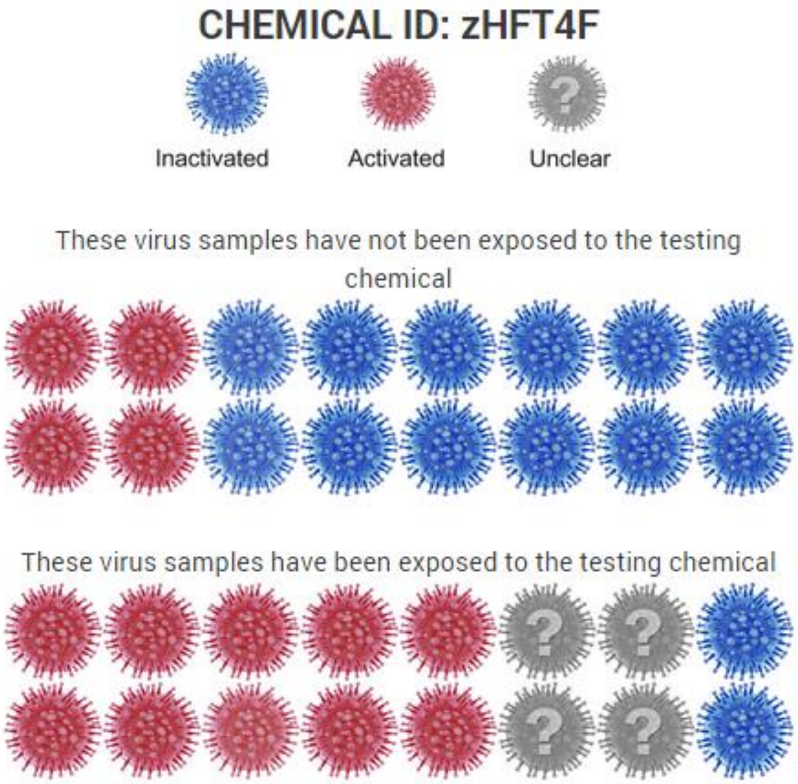

Omission positive contingency condition

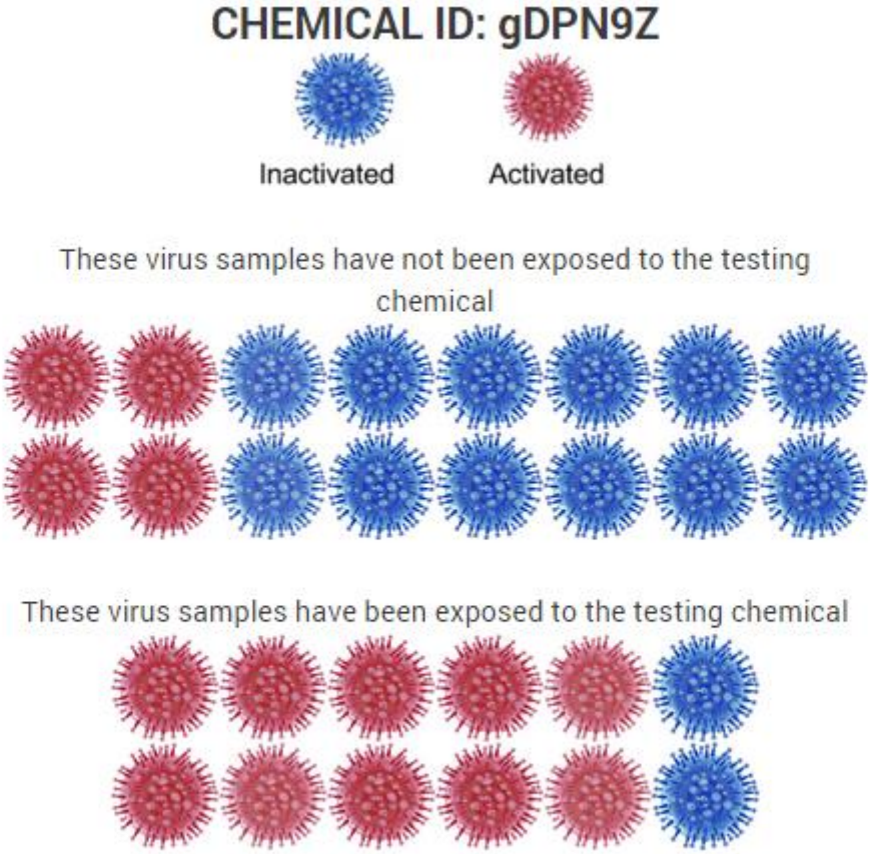

Experiment 2 and 3 Causal Reasoning Task - High Cognitive Demand

The instruction of scenario and measurement questions were the same as Experiment 1.

Example stimuli

There were five different types of paired stimuli.

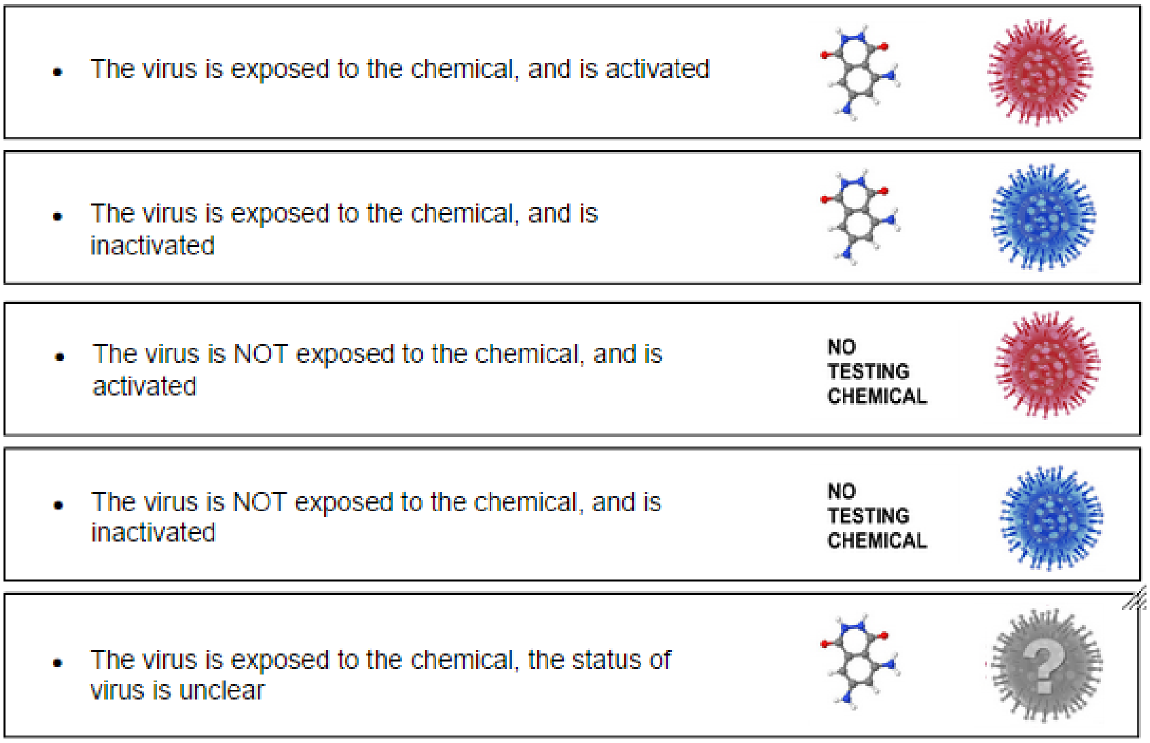

An example of the frequencies of the paired stimuli in the zero contingency condition (PC condition) are shown below. The total 32 pairs were randomized presented in the experiment.

Type	Paired Stimuli	Frequency
Chemical Present & Effect Present	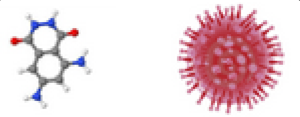	10
Chemical Present & Effect Absent	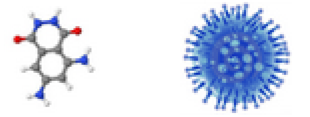	2
Chemical Absent & Effect Present	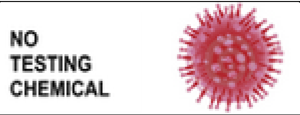	12
Chemical Absent & Effect Absent	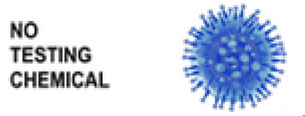	4
Chemical Absent & Effect Unknown	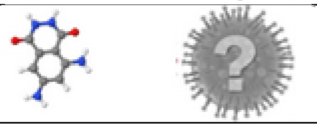	4

Experiment 3 Causal Reasoning Task - Low Demand Condition

The instruction of the evaluation scenario, the frequencies of stimuli in different conditions, and the judgment questions were the same as the high demand condition. The presentation of the evidence in the low demand condition in Experiment 3 was the same as the one in Experiment 1.
