# Supplementary material for: Adapting to an Uncertain World: Cognitive Capacity and Causal Reasoning with Ambiguous Observations
Source: PLoS One. 2015 Oct 15;10(10):e0140608. doi: 10.1371/journal.pone.0140608 (PMC4607167; doi:10.1371/journal.pone.0140608)
Supplement: S2 Appendix — (PDF) [file pone.0140608.s002.pdf]

S2 Appendix. Introduction of Beta GLM and Model Syntax

The ordinary linear models such as ANOVA assume that the dependent variable is normally distributed, and has unbounded support. However, the dependent variables in the majority of causal reasoning studies are double bounded (such as numerical or probability ratings from 0 to 100, or 0 to 100%). When a causal strength is approaching extreme values such as 0 or 100, the distribution of the causal ratings can be highly skewed. The change in the distribution shape can also result in the inconsistent variance of the dependent variables. Thus, both the assumption of normality and homogeneity of variance are violated. In addition, the ordinary linear models only focus on the mean, while being unable to capture the change in the variability or dispersion of the dependent variable. The dispersion of a dependent variable can be an indicator of the extent to which subjects agree in perceiving the causal relationships. The higher dispersion in the dependent variable suggests a greater discrepancy among subjects in providing responses. An alternative method is beta generalized linear model (GLM) based on the beta distribution. The beta GLM allows for more flexibility in the shape of the variable distribution and allow the assumption of normality being relaxed.

The Beta GLM

Suppose that $y \sim \mathrm{Beta}(\alpha, \beta) \in (0,1)$, where $\alpha, \beta > 0$ are two shape parameters. The density function of y is

$$f(y|\alpha, \beta) = \frac{\Gamma(\alpha + \beta)}{\Gamma(\alpha) + \Gamma(\beta)}\, y^{\alpha-1}(1-y)^{\beta-1}, \alpha > 0, \beta > 0 \qquad (1)$$

where $\Gamma(\bullet)$ denotes the gamma function. Different values of α and β result in different distribution shapes such as skewed uni-modal, uniform and U-shaped. The location parameter and dispersion parameter of y are

$$\mu = E(y) = \frac{\alpha}{\alpha + \beta}$$

$$\sigma^2 = Var(y) = \frac{\alpha\beta}{(\alpha + \beta)^2(\alpha + \beta + 1)}$$

Let $\phi = \alpha + \beta$. For a fixed μ, the dispersion $\sigma^2 = \mu(1 - \mu)/(1 + \phi)$ decreases as ϕ increases, so ϕ is known as a precision parameter. Replacing α and β with μ and ϕ, a re-parameterised function is obtained:

$$y \sim \text{Beta}(\mu\phi, \phi(1 - \mu)) \quad (2)$$

$$f(y|\phi, \mu) = \frac{\Gamma(\phi)}{\Gamma(\phi\mu) + \Gamma(\phi(1 - \mu))} y^{\phi\mu - 1}(1 - y)^{\phi(1-\mu)-1} \quad (3)$$

whereas $f(y|\phi, \mu)$ gives the probability of observing y_i given μ and ϕ, a likelihood function presents the probability of μ and ϕ given the set of observations of *y*. The log-likelihood of μ and ϕ given y_i is

$$\log L(\mu, \phi|y_i) = \log\Gamma(\phi) - \log\Gamma(\mu\phi) - \log\Gamma(\phi - \mu\phi) + (\mu\phi - 1)\log(y_i) + (\phi - \mu\phi - 1)\log(1 - y_i) \quad (4)$$

Let $\boldsymbol{X}$ and $\boldsymbol{W}$ be two covariate matrices associating with the location and precision of *y*, respectively. Their row vectors $\boldsymbol{x_i}$ and $\boldsymbol{w_i}$ are the *i*th independent observations, while column vectors X_k and W_k are the *k*th IV. The linear combinations of $\boldsymbol{X}$ and $\boldsymbol{W}$ are $\boldsymbol{\eta_1} = \boldsymbol{X\beta}$ and $\boldsymbol{\eta_2} = \boldsymbol{W\delta}$, where $\boldsymbol{\beta}$ and $\boldsymbol{\delta}$ are two vectors of parameters. Let us assign value of 1 to elements in X_0 and W_0, so that β_0 and δ_0 are two intercepts in the regression sense.

Two link functions $f(\mu)$ and $g(\phi)$ are used to relate $\boldsymbol{\eta_1}$ and $\boldsymbol{\eta_2}$ with $\boldsymbol{\mu}$ and $\boldsymbol{\phi}$ of *y*. In the beta GLM, $\boldsymbol{\mu}$ is positive on the interval (0, 1) and $\boldsymbol{\phi}$ is also positive, while $\boldsymbol{\eta_1}$ and $\boldsymbol{\eta_2}$ might not be. Therefore, $f(\mu)$ and $g(\phi)$ must satisfy the restrictions on y. In this paper, a logit link is used to link to link μ and $\boldsymbol{\eta_1}$ because the logit link can restrict $\boldsymbol{\eta_1}$ in

(0, 1). In addition, $\boldsymbol{\beta}$ can be interpreted as odds ratio with a logit link, as this allows for a straightforward interpretation of the importance of any β.The logit link is

$$f(\mu_i) = \eta_{1i} = \log\left(\frac{\mu_i}{1-\mu_i}\right) = \boldsymbol{x_i}\boldsymbol{\beta} \tag{5}$$

The precision parameter $\boldsymbol{\phi}$ can be linked with $\boldsymbol{\eta_2}$ via a log link, which can constrain $\boldsymbol{\eta_2}$ to be positive. The log link is given by

$$h(\phi_i) = \log(\phi_i) = \boldsymbol{w_i}\boldsymbol{\delta} \tag{6}$$

Equation 5 and 6 form the basis of a beta GLM. The location submodel describing how predictors explain the mean of the DV is defined by $f(\mu)$ with $\boldsymbol{\delta}$ being held constant. Similarly, a dispersion submodel, which represents how IVs explain the precision parameter of the DV, is defined by $h(\phi)$ with $\boldsymbol{\beta}$ being held constant.

Example

In our paper, we examined the effects of ambiguity on causal ratings in Experiment 2. The response values ranged from -100 to 100. The responses were first linearly transformed into the [0, 1] interval, via y_i=((Y_i+100)*(N−1)/200+0.05)/N, where *Yi* is the raw causal rating, *N* is the sample size. Predictors included the ambiguity conditions, contingency conditions, *n*-back scores and Ospan scores.

Dummy variables for the two experimental factors were produced by using the effect coding. Thus the intercepts of the two submodels indicate the mean and overall precision. Two dummy variables: *C2* and *C3* were used to code the three contingency conditions. Both *C2* and *C3* were coded -1 for subjects' ratings in the positive contingency condition. *C2* was coded as 1 for the ratings in the zero contingency condition, and as 0 for the negative contingency condition. Likewise, *C3* was coded as 0 for the ratings in the zero contingency condition, and as 1 for the negative contingency

condition. The dummy variable *Ambiguity* was coded as -1 for ratings in the unambiguous condition, and 1 for the ratings in the AU condition.

Table 4 in the article displays the estimated coefficients for the main effect model. The first half of Table 4 outlines the estimated coefficients for the location submodel. The intercept of the location submodel indicated the overall mean of the causal ratings. The value can be converted back to the actual responses via reverse the link function of the location submodel $y_i = exp(0.16)/(1+exp(0.16)) = 0.54$ and, $Y_i = y_i * 200 - 100 = 7.89$. It should be noticed that an intercept valued at 0 is equivalent to the rating at 0 on the actual causal rating scale. The intercept has a 95% confidence interval at [0.09, 0.24], which means the overall mean ratings of subjects were significantly higher than zero.

A positive coefficient in the location submodel indicates a positive relationship between the predictor and the mean of the dependent variable. Because we used the effect coding, the coefficient value for *C2* indicates the difference between subjects' ratings in the zero contingency condition and the overall mean. And the coefficient value for *C3* indicates the difference between subjects' ratings in the negative contingency condition and the grand mean.

According to Table 4, subjects' ratings in the zero contingency condition were significantly different from the overall mean, $b_2 = 0.12$. This also suggests that subjects' causal ratings in the zero contingency condition were significantly higher than zero. Subjects' ratings in the negative contingency condition were substantially lower than the overall mean, as suggested by $b_3 = -0.96$.

On the other hand, the 95%CI of the coefficient of *Ambiguity* contains 0, indicating that the causal ratings in the AU condition were not significantly different

from the ratings in the unambiguous condition. Similarly, the coefficient results of the two WM measures suggests that there was no significant association between the performance of the two WM measures and the causal ratings.

The second half of Table 4 shows the results of the precision submodel. A positive coefficient indicates a greater precision, or smaller variance of the causal ratings. According to Table 4, the ratings of the negative contingency condition had significantly greater variability than the overall ratings of the all three conditions (d_3 =-0.32). In addition, both *n*-back scores and Ospan had positive associations with the precision of the causal ratings, d_4 = 0.10 and d_5 = 0.09, respectively. This suggests that subjects with higher WM scores had greater homogeneity in their causal ratings than those who had lower WM scores. Finally, there was a significant interaction between the ambiguity conditions and Ospan. In the unambiguous condition (i.e., *Ambiguity* = -1), Ospan was positively associated with the precision of the causal ratings $d = d_5 + (-1)*d_6 = 0.09 – (-1)*(-0.16) = 0.25$. On the other hand, in the AU condition (i.e., *Ambiguity* = 1), Ospan had negative association with the precision of the causal ratings $d = d_5 + (1)*d_6 = 0.09 – (1)*(-0.16) = -0.07$.

BUGS model syntax

```
model{
for (i in 1:N) # for i in N response
      {
      # y is the response value that follows a beta distribution
      y[i]~dbeta(a[i],b[i])

      #Re-parameterize the distribution by using the mean (mu)
and precision (phi) parameters
      a[i]<-  mu[i]*phi[i]
      b[i]<-(1-mu[i])*phi[i]

      #Predictors are then linked by link functions
      #C2 and C3 are dummy variables for contingency conditions
      #amb is the dummy variable for ambiguity conditions
      #nback and ospan are the two WM scores
      logit(mu[i]) <- b0 + b1* C2[i]+ b2 * C3[i] + b3 * amb[i]
                   + b4 * nback[i]+ b5 * ospan[i] + u[j]
      log(phi[i]) <- d0 + d1* C2[i]+ d2 * C3[i] + d3 * amb[i]
         + d4 * nback[i]+ d5 * ospan[i] + d6 * amb[i] * ospan [i]
}

for (j in 1:J)
#the random intercept for each of the J participants
{u[j] ~ dnorm(0,tau) }

#Prior distributions for coefficients
b0 ~dnorm(0.0, 1.0E-6)
b1 ~dnorm(0.0, 1.0E-6)
b2 ~dnorm(0.0, 1.0E-6)
b3 ~dnorm(0.0, 1.0E-6)
b4 ~dnorm(0.0, 1.0E-6)
b5 ~dnorm(0.0, 1.0E-6)
d0~dnorm(0.0, 1.0E-6)
d1~dnorm(0.0, 1.0E-6)
d2~dnorm(0.0, 1.0E-6)
d3~dnorm(0.0, 1.0E-6)
d4~dnorm(0.0, 1.0E-6)
d5~dnorm(0.0, 1.0E-6)
d6~dnorm(0.0, 1.0E-6)

tau <- exp(2*lsu)
lsu~dnorm(-0.5, 1.0E-6)
}
```
